# Life-Stage Dependent Plasticity in the Auditory System of a Songbird Is Signal and Emitter-Specific

**DOI:** 10.3389/fnins.2020.588672

**Published:** 2020-12-04

**Authors:** Nicolas M. Adreani, Pietro B. D’Amelio, Manfred Gahr, Andries ter Maat

**Affiliations:** ^1^Department of Behavioural Neurobiology, Max Planck Institute for Ornithology, Pöcking, Germany; ^2^Konrad Lorenz Research Center, University of Vienna, Grünau im Almtal, Austria; ^3^FitzPatrick Institute of African Ornithology, University of Cape Town, Rondebosch, South Africa

**Keywords:** auditory plasticity, secondary auditory area, zebra finch, individual recognition, caudomedial nidopallium, context dependent

## Abstract

Social animals flexibly use a variety of vocalizations to communicate in complex and dynamic environments. However, it remains unknown whether the auditory perception of different vocalizations changes according to the ecological context. By using miniature wireless devices to synchronously record vocal interactions and local neural activity in freely-behaving zebra finches in combination with playback experiments, we investigate whether the auditory processing of vocalizations changes across life-history stages. We show that during breeding, females (but not males) increase their estrogen levels and reply faster to their mates when interacting vocally. These changes are associated with an increase in the amplitude of the female’s neural auditory responses. Furthermore, the changes in auditory response are not general, but specific to a subset of functionally distinct vocalizations and dependent on the emitter’s identity. These results provide novel insights into auditory plasticity of communication systems, showing that the perception of specific signals can shift according to ecologically-determined physiological states.

## Introduction

In vocal species where different messages are conveyed by multiple signals, the receiver brain needs to be capable of categorizing such signals (Miller et al., 2003; Prather et al., 2009). This can be combined with mechanisms that enhance the saliency of the signals in a given context, such as vocal plasticity in the senders or auditory plasticity in the receivers (Shannon, 2001; Bergstrom and Rosvall, 2011; Bradbury and Vehrencamp, 2011). Investigating how these mechanisms interact with signal categorization is central to our understanding of the coevolution of production and perception of complex vocal communication systems.

To optimize communication in changing contexts senders can modify the frequency, amplitude, timing or type of vocalizations to adjust to environmental changes or address specific receivers (Schwartz, 1987; Hotchkin and Parks, 2013; Gill et al., 2015; Pomberger et al., 2018), and receivers can undergo changes in their auditory system (Sisneros et al., 2004; Feinberg et al., 2006; Arch and Narins, 2009; Brenowitz and Remage-Healey, 2016). For example, female plainfin midshipman fish (*Porichthys notatus*), that use phonotaxis to find their mates, go through seasonal and hormone-mediated changes in the peripheral auditory system that enhance their ability to detect males (Sisneros et al., 2004). Although life-stage dependent auditory plasticity was investigated in diverse species (Sisneros et al., 2004; Arch and Narins, 2009; Caras, 2013; Brenowitz and Remage-Healey, 2016), it has never been addressed in relation to multiple functionally distinct vocal signals. Most research on life-stage auditory plasticity has focused either on relatively simple courtship signals in fish and anurans (Bass et al., 2008; Arch and Narins, 2009) or exclusively on songs in seasonally breeding birds [reviewed by Brenowitz and Remage-Healey (2016)]. Thus, whether life-stage dependent auditory plasticity is specific only toward certain or toward every component of a repertoire, remains unknown.

**FIGURE 1:**
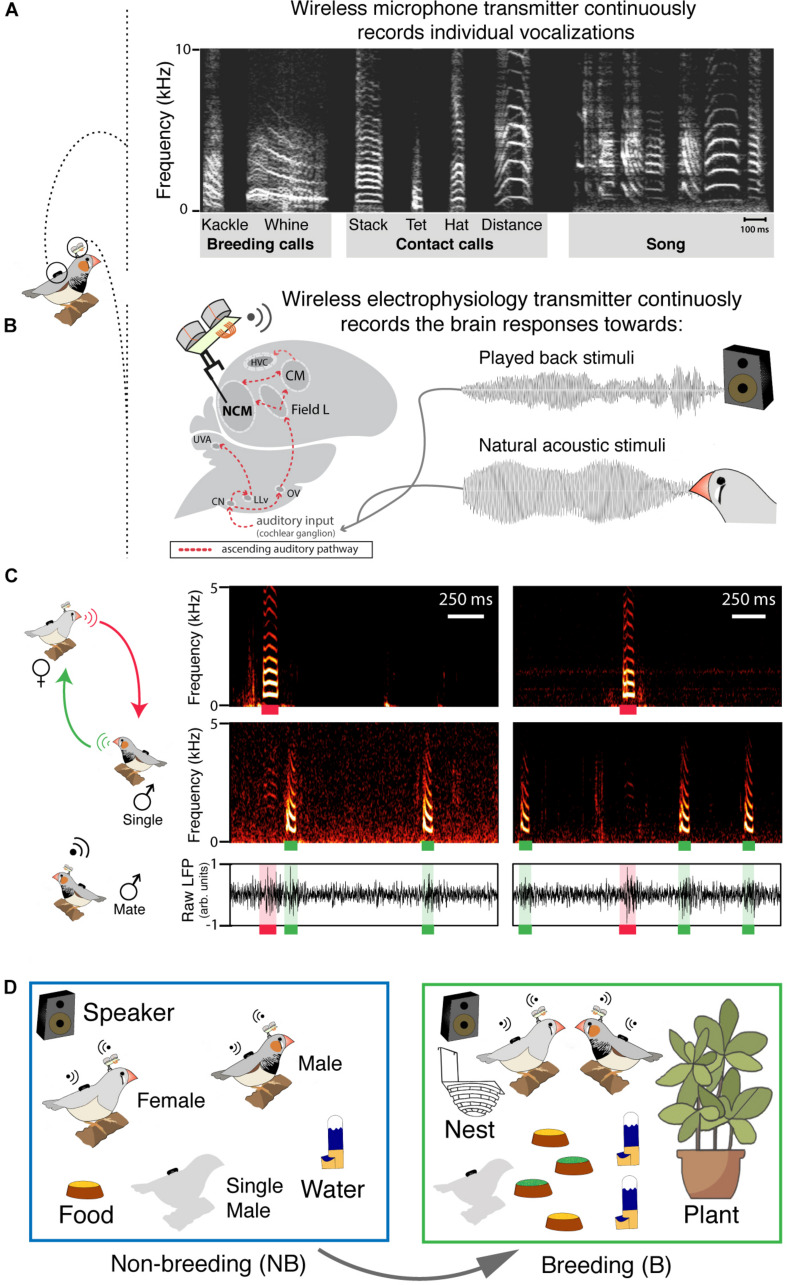
Synchronized individual recordings of vocal and neural activity in group-living birds behaving freely. **(A)** Sonogram of the typical zebra finch repertoire: contact and breeding calls are produced by both sexes but only males sing. **(B)** Chronically implanted electrodes record from the caudomedial nidopallium (NCM); the ascending auditory pathway is shown in red (CN, cochlear nucleus; LLv, ventral lateral lemniscus; UVA, nucleus uvaeformis; Ov, nucleus ovoidalis; CM, caudal mesopallium; HVC, proper name). **(C)** Example of synchronous recording of a vocal interaction and NCMs neural responses within an experimental trio. The top, middle and bottom panels correspond, respectively, to vocalizations of the female (red), vocalizations of the extra-pair male (single; green) and the paired-male’s NCM LPF activity. Raw LFP corresponds to the 300 Hz threshold low pass filtered local field potential (LFP) response in NCM. **(D)** Sketch of non-breeding (NB) and breeding (B) life stages.

Songbirds can be highly social and learn vocalizations (Fitch, 2000). Further, they also produce multiple innate vocalizations, which represent the most common and ancestral vocalizations across vertebrates (Marler, 2004). Thus, songbirds provide excellent models for investigating how auditory plasticity relates to vocal communication with different acoustic signals. Zebra finches are opportunistic breeders with a diverse repertoire that includes song and different innate vocalizations classified broadly as contact and breeding calls (Figure 1A; Zann, 1996). Both contact and breeding calls carry information on the identity of the emitter that allow for individual recognition (D’Amelio et al., 2017a; Elie and Theunissen, 2018). These calls are used in a turn-taking manner (D’Amelio et al., 2017b), a vocal strategy that is also used by some primates, including humans (Flack, 2013). Within female-male dyads, contact call interactions are plastic and important for pair formation and bond maintenance (Zann, 1996; Gill et al., 2015; D’Amelio et al., 2017b). Likewise, breeding-call interactions are used for within-pair coordination during reproduction (Boucaud et al., 2017). Also, the call combinations used change drastically depending on the life stage (Gill et al., 2015). Whether this plasticity of vocal interactions is coupled with plasticity in auditory processing is unknown, as is whether the degree of auditory plasticity varies across signal categories (i.e., call types) and sender identity.

We investigated breeding-induced plasticity in the *caudomedial nidopallium* (NCM), an auditory area of the songbird brain analogous to the mammalian auditory cortex, and involved in high-order acoustic processing (Chew et al., 1996; Theunissen and Shaevitz, 2006; Figure 1B). NCM has composite receptive fields (Kozlov and Gentner, 2016) that encode the different vocal categories (Elie and Theunissen, 2015) as well as familiarity of acoustic stimuli (Lu and Vicario, 2017). Further, estrogen, which in breeding females increases drastically both in circulation and in the brain (Johnson, 2014), can also be rapidly modulated in the brain of males (Remage-Healey et al., 2008, 2010). Estrogen modifies NCMs’ local spiking activity and affects the activity of vocal production regions (Remage-Healey and Joshi, 2012). In our study, we focused on NCMs’ local field potential (LFP) activity. LFPs are the low frequency signals (2–200 Hz) that result from combined neuronal electrical activity (Mitzdorf, 1985; Buzsáki et al., 2012). Within the LFPs different frequency bands can be functionally distinguished, for instance, according to their role in regulating the excitability of neural networks (Buzsáki et al., 2012). The low gamma-band (30–60 Hz), in particular, can represent the relation between excitatory and inhibitory neuronal populations (Buzsáki et al., 2012).

In freely behaving captive birds and in combination with playback experiments, we tracked NCMs’ LFP activity in synchrony with vocal behavior (Figure 1C) while inducing a transition from non-breeding to breeding context (Figure 1D). First, we tested whether breeding-induced changes in vocal dynamics co-occurred with changes in the auditory system of both birds within a reproductive pair. Further, in combination with a playback experiment, we tested whether the change in the auditory responses induced by the change in context represents a general change in the auditory system or whether it is specific for certain vocalization types. Finally, because zebra finch’s vocal signals carry information of the emitter (D’Amelio et al., 2017a; Elie and Theunissen, 2018), we tested whether auditory changes were emitter-specific. Within the neural sciences, longitudinal experiments implementing electrophysiology in animals behaving freely in social groups have always been a great challenge (Krakauer et al., 2017). We achieved this and combined it with playback experiments to provide novel insights into the interaction between sensory plasticity, context and signal categories.

## Materials and Methods

### Experimental Model and Subject Details

#### Birds

We used a total of 36 adult, parent-raised zebra finches (24 males, 12 female) that were kept on a 12/12-h light/dark cycle with food and water *ad libitum*. The birds were separated into groups of three, named trios, after maturity (ca. 6 months old). Each group was composed of two males and one female. The unbalanced sex ratio allowed for flexibility of choice in the formation of the pair, and for moderating forced relationships. Eight trios were used for the experiment without electrophysiology and six for the NCM-implant experiment. Every trio was kept visually isolated from each other. The trios were kept together for a period of at least 3 months in which a pair formed and bred successfully once. The unpaired male was named “single.” The paternity of the chicks was confirmed molecularly and we never had a case of extra-pair paternity. Thus, we are confident of a stable social structure within each trio.

### Method Details

#### Non-implanted Breeding Onset Experiment - Effect of Breeding Onset on Vocal Behavior

First to study the influence of the environmental change on the vocal behavior we performed the experiment with birds that carried microphone transmitters but were not implanted with electrophysiology transmitters. For each trio, 4 to 5 days prior to the beginning of the recordings, the birds were equipped with microphone backpacks and moved in a visually and acoustically isolated aviary (1 × 1 × 1 m) equipped with an external microphone (TC20, Earthworks, United States) and a loudspeaker (KENWOOD KFC-1761S, Kenwood Electronics, London, United Kingdom). The microphone transmitters have been already used in previous studies and a detailed description and validation of the method is available in Gill et al. (2016). Briefly, this microphone transmitter allows for precise, continuous and individual vocal recording of the carrier bird. Additionally, the setup has a system-based synchrony between transmitters’ signals enabling the study of fast “turn-taking” vocal interactions. Before the experiment the birds had 4 to 5 days of habituation and acclimation, which is enough to recover normal vocal and motor activity (Gill et al., 2016). After acclimation the birds were recorded during the next 8 days for 4 h every day after “lights on.” During the first 3 days the birds were kept in a stimulus-poor environment with only water and food. On the evening of the third day multiple varieties of food were given. In addition, plants, nest sites and nesting material were provided, and these enriched conditions were maintained for the remaining 5 days. This change of condition led to breeding related behaviors of the pair such as nest inspection and nest building. We therefore named this time point: “breeding onset.”

#### NCM-Implant Experiment - Effect of Breeding Onset on Auditory Responses in NCM During Vocal Interactions

In order to investigate the changes in auditory responses after the breeding onset, we repeated the procedure described in the previous section. During this set of experiments the mated birds had an electrode implanted in the right *caudomedial nidopallium* (NCM), a secondary auditory area, and carried an electrophysiological transmitter (Figures 1A,B). Auditory processing in NCM has been shown to be lateralized (e.g., Phan and Vicario, 2010). Since implanting electrodes simultaneously in both hemispheres of NCM was technically impossible to achieve with our telemetric technique, we selected the right hemisphere because it is the one with higher absolute response strength and faster adaptation toward conspecific vocalizations in both sexes (Phan and Vicario, 2010; Yang and Vicario, 2015). We used six trios and only the established couple was implanted in each group. The day after the implantation was excluded from behavioral scoring as birds need one day to recover from the surgery (Schregardus et al., 2006). In this way we recorded NCMs activity within 3 days of non-breeding context and 4 days of breeding context. After implantation, simultaneous and synchronized recordings of the audio and electrophysiological transmitters occurred every day during the 4 h after the lights turned on. The electrophysiological and vocal signals are digitized by the same device (M-audio M-Track Eight, United States) and processed with a multi-channel ASIO-based software recording all tracks with minimal latency and synchronously. When the experiment ended, electrolytic lesions were performed through the recording electrodes, the birds were euthanized, and brains were extracted and stained using standard Nissl method to confirm electrode position (representative pictures are provided with the articles’ data).

##### Surgical procedure and transmitting device

The surgical procedures of each couple were performed between 7 and 12 a.m. A detailed description of the surgery and the transmitter can be found in Schregardus et al. (2006), and Ter Maat et al. (2014). Briefly, the birds were anesthetized with isoflurane inhalation (1–1.8% at 0.5 l O2/min). Next, the birds were wrapped in a small blanket and placed in a stereotaxic head. During the entire procedure the birds’ temperature was controlled and maintained constant at 38°C degrees with a heating pad. For the implantation a craniotomy was performed over the bifurcation of the mid-sagittal sinus without opening the second layer of the skull and this was used as a reference point. Using stereotaxic coordinates (*x* = 500 μm, *y* = 500 μm with a head angle of 45°) a second and complete craniotomy was done over the right *caudomedial nidopallium* (NCM). Later, a 2 MΩ tungsten electrode of 5 mm in length (Frederic Haer Company, FHC, United States) was inserted at depths of 600–800 μm into NCM (Figure 1B). For the reference electrode we used a platinum wire (0,025 mm, Goodfellow), which was implanted between the bone layer and the dura mater. Both electrodes were fixed to a custom-made connector to which the electrophysiology radio transmitter was connected before releasing the bird back into the experimental aviary. The input circuit of the radio transmitter is equipped with a high-pass filter (−3 dB at 50 Hz) and has no amplification. Further, the receiver (AR8600, AOR, Japan) has attenuation in the low frequencies (12 dB attenuation at 10 Hz).

#### NCM-Implant Night Playback Experiment – Effect of Breeding Onset on NCM Auditory Responses During Playback

During the day, the auditory system is constantly activated by multiple acoustic stimuli (vocal and non-vocal) while simultaneously interacting with the other sensory systems. To investigate breeding induced changes that were specific to the auditory system alone (i.e., minimizing the interaction with other sensory inputs), we performed playbacks during the night to obtain well-defined and clean auditory responses (e.g., Figure 3A). The playbacks took place during two nights during the non-breeding context and two nights during breeding context between 12 pm and 2 am. In order to emulate more naturalistic settings we performed our experiment in big aviaries. It was, therefore, unpredictable where the birds would roost. Thus, it was impossible to video record the birds during the night to precisely determine whether they were asleep or not. However, the period during which we performed our playbacks was when arousal probability is at its minimum (Szymczak et al., 1996). We analyzed the auditory response of six birds, three males and three females. We used a speaker driven at 65–70 dB measured at 1 m (SPL meter, HD600; Extech, Nashua, NH, United States). Every vocalization used as stimulus was recorded from a central microphone during the habituation phase. Before the playbacks the amplitude of every sound was normalized to −5 dB maximum (Amadeus Pro, HairerSoft, Kenilworth, United Kingdom). The entire repertoire was played (i.e., six vocalization types excluding hat calls; Figure 1A) of the male and female of each couple and white noise as a control. In order to study whether the familiarity could play a role in life-stage auditory plasticity, we also played back different call types recorded from different emitters that differed in the social relevance toward the focal female: from an unfamiliar male; the familiar single male, an unfamiliar female and the birds’ own vocalization. Every acoustic stimulus was repeated 30 times and the playbacks were pseudorandomized along the trial. Specifically, the sounds that were used were: stack calls, tet calls, distance calls, kackle calls and whine calls of males and females; song from males, and a white noise internal control of 100 ms length.

#### Blood Sampling and Estrogen Quantification– Effect of Breeding Onset on Estrogen Levels

Both in non-implanted and NCM-implanted birds three blood samples were obtained during the experiments. One the day before the beginning of the experiment (the day of the brain surgery in the case of the NCM-implanted birds), a second 24 h after the breeding onset and a third 24 h after the last day of experiment. 65 μL of blood were extracted from the wing vein of each bird within 10 min after the experimenter entered the room.

Immediately after extraction, samples were centrifuged at 3000 rpm for 10 min. Plasma was extracted and promptly frozen at −80°C. The estrogen 17β-estradiol (E2) was extracted from the plasma and concentrations were determined by radioimmunoassay following the procedures described by Goymann et al. (2008). E2 concentration was obtained from two separate assays one for males and another for females. The mean recovery (±sd) was 61% (±5%) and the lower detection limit was 2.2 pg/ml. The intra-assay variation was calculated from an extracted chicken pool and was 30%.

### Quantification and Statistical Analysis

#### Vocal Activity and Interaction

Every recording was done at a sample rate of 44100 Hz. Before the analyses, the sound files were processed semi-automatically through a custom written software to cluster the individual vocalizations into the different call types and song (Figure 1C; Zann, 1996; Gill et al., 2016; D’Amelio et al., 2017a,b). With this software we obtained the total number of vocalizations for each call type that each bird emitted during the 4 h of recording every day. In some cases vocalizations from other birds appeared in the recording, but they were clearly distinguishable and vocalizations were never wrongly assigned (Gill et al., 2016). Additionally, we also obtained the time stamp of every vocal event. Using the software R (R Core Team, 2013) we subsequently generated a unified dataset containing the following information: vocalization type, time of onset, day and emitter. Given that all the recordings were perfectly synchronized ^54^ we could use the time information of every call to calculate the parameters that were then used to describe the vocal interaction between birds: reply strength and latency of response.

The reply strength (RS) has already been used for describing the vocal interaction of zebra finches (Ter Maat et al., 2014; Gill et al., 2015) and reflects the intensity with which a bird replies to another relative to its baseline calling activity. We designated a time window of four seconds before the onset of the vocalization and four seconds after with a bin width of 50 milliseconds. Then, given an interaction the reply strength is calculated as:

(1) $$R\,S=\frac{\left(N\,r\,e\,s\,p\,o\,n\,s\,e-N\,b\,a\,s\,e\,l\,i\,n\,e\right)}{N\,r\,e\,s\,p\,o\,n\,s\,e+N\,b\,a\,s\,e\,l\,i\,n\,e}$$

where *Nresponse* is the total number of calls that bird 1 elicited after the calls of bird 2 during the first 500 milliseconds after the onset and *Nbaseline* is the total number of calls that bird 1 elicited during the first 500 milliseconds before the onset.

We define latency as the time gap between a vocalization and the answer of a second individual. Every call within 500 milliseconds of the onset of the reference call was considered an answer (Ter Maat et al., 2014). Using the time stamp of every vocalization the average reply latency (RL) was calculated for the different call interactions.

In our study we calculated RS and RL for every symmetrical call interaction (i.e., both birds use the same call type to address each other, for example stack interaction, tet interaction, etc.) and the two most common asymmetrical interactions (tet-stack interaction, stack-tet interaction).

#### Local Field Potential Analyses

For the LFP analysis during the day the calls were classified in 3 categories: “isolated call” (if there was no other sound 5 s before or after the call), “answered by the focal individual” if there was an answer within 0.5 s previous to the call) and “answer to the focal individual” (if it was answered within the next 5 s after the call). The time stamps and durations of each call type were used to cut the wave files containing the neuronal activity (defined as the stimulus fragment, Figure 3A) and the wave fragments prior to the vocalization (defined as the baseline fragment, Figure 3A). Using the function “*butter*” of the package “*signal*” (Signal Developers, 2013), a 1st order Butterworth filter was applied for frequencies from 10 to 200 Hz with bands of 10 Hz. Subsequently, for each band the root mean square (RMS) was calculated using the function “*rms*” of the package “*seewave*” (Sueur et al., 2008). The time window for which the RMS was computed corresponded the total duration of each vocalization. The same time window was used for the calculation of both for the baseline and the stimulus RMS. After this, we subtracted the value of RMS during the baseline to the value of RMS during the stimulus and defined the result as the “LFP Auditory Response Strength” (Hereafter ARS; Figures 3A,B). The LFP levels at rest did not influence our analysis because our measure (ARS) was relative to the baseline and for the night playbacks comparisons were made with the internal controls. For all our analyses, the ARS was normalized between −1 and 1 within each bird.

For the LFP analyses during interactions we used the stack and tet contact calls because these were produced the most by every bird.

#### Statistical Analyses

All the statistical analyses were performed in R (R Core Team, 2013), we ran linear mixed models under a pseudo-Bayesian framework using the packages “*lme4*” (Bates et al., 2014), “*arm*” (Gelman et al., 2015), except when general additive mixed models were applied using the package “*mgcv*” (Wood, 2011). For every linear model (package “*lme4*”) the restricted maximum likelihood estimation method was applied and all the assumptions were checked by visual inspection of the residual plots. Specifically, we checked that the residuals were independent and identically distributed verifying that the residuals were normally distributed and that the residual variance was approximately consistent for the entire range of fitted values. Subsequently we used the “sim” function, package “arm,” to draw 10000 times random values from the joint posterior distribution of the model parameters. The drawing used flat priors for models’ parameters estimation, defined as p(β) ∝ 1 (a horizontal line at 1) for the model coefficients and p(σ^2^) ∝ 1/σ^2^ for the variance parameters. We used the 10000 estimated parameters to extract the 95% credible interval (CrI) around the mean (Gelman and Hill, 2007), representing the uncertainty around our estimates. In all figures we display the predicted coefficients estimate from the models’ and the 2.5–97.5% CrI. We considered an effect to be statistically meaningful when the probability of the difference between the compared mean estimates was higher than 95% [for further details on statistical inference see (Korner-Nievergelt et al., 2015)].

##### Total vocalizations

For the total number of breeding calls analysis, kackle and whine calls were considered together (Supplementary Figure 1). Breeding calls were modeled using a linear mixed model with gaussian link function after applying a logarithmic transformation to achieve normality distribution of the residuals. The model explanatory variables treatment, trial, sex and their interactions were categorical. Being treatment: non-breeding (NB) vs. breeding (B); trial: implanted vs. non-implanted couples; sex: male and female. In order to account for the individual variation “ID” was included as a random slope within treatment. CrIs and fitted values of the model with were plotted in Supplementary Figure 1. For the exact fitted values and CrIs see Supplementary Table 1.

##### Estrogen levels

**FIGURE 2:**
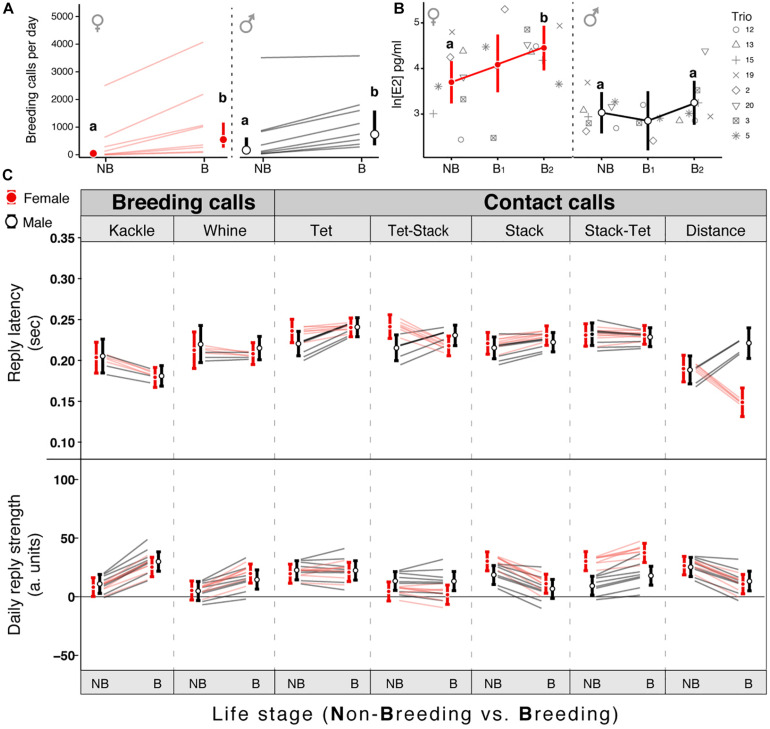
Onset of breeding conditions affects the call-based vocal interactions. **(A)** Number of breeding calls elicited per morning by males (black) and by females (red) during non-breeding (NB) and breeding (B) conditions. **(B)** Change in estrogen (E2) levels of males and females from NB to B (B_1_: 24 h after the breeding onset; B_2_: 24 h after last experimental day). **(C)** Breeding induced change in reply latency (top) and reply strength (bottom) of males (black) and females (red) for seven different call interactions. Circles reflect the mean estimate from the model, vertical bars the 95% credible intervals (CrI) and each line represents the change within an individual bird. In panels **(A,B)** Different letters indicate a posterior probability of difference higher than 95%. For panel **(C)** a probability of difference higher than 95% can be assumed if the estimates of a group do not overlap the CrI of the other group.

Estrogen levels were modeled with two linear mixed effect models, one for the non-implanted group and another for the NCM-Implanted one. For both the outcome variables was the logarithmic transformation of the estrogen concentration. The explanatory variables were sex (Male or female), treatment (NB, B_1_, and B_2_) and their interaction. To account for the repeated measures and the between-trio variation ID was nested within trio as random factors. CrIs and fitted values of the models were plotted in the Figure 2B and Supplementary Figure 4. For the exact estimates and CrIs see Supplementary Table 2.

##### Reply latency and reply strength

Both reply strength (Supplementary Figure 2) and reply latency (Figure 2B and Supplementary Figure 3) were modeled with linear mixed models. The explanatory variables for both models were: treatment, trial, relation and call exchange and the interactions between these. Being treatment: non-breeding (NB) and breeding (B); trial: implanted and non-implanted couples; relation: “female to male” and “male to female”; and call exchange: the most common interactions (as described in section “Non-Implanted Breeding Onset Experiment - Effect of Breeding Onset on Vocal Behavior”). As in the models for the total amount of calls the random factors “day” and “experimental unit” were added. CrIs and fitted values of the models were plotted in the Supplementary Figures 2, 3. For the exact estimates and CrIs see Supplementary Table 3.

##### Local field potential (LFP)

###### Auditory response and selection of the LFP-band of interest

In order to investigate whether our LFP measures were auditory responses we performed one linear mixed model of the calculated ARS for each individual bird. The outcome variable for every model for daytime interactions was the ARS (transformed as: Log(*x*^2^); where “*x*” is the ARS). The explanatory variable was the LFP band (10–200 Hz) and we included the random factor “call information” to account for the different contexts of the acoustic stimuli (isolated, reply and replied). The results for every bird analyzed are depicted in Supplementary Figure 5 and the detailed estimates are in Supplementary Table 4. Taking a similar approach as (Lewandowski and Schmidt, 2011), we defined our band of interest as the bands with the highest ARS. These were the low gamma bands 30, 40, and 50 Hz (hereafter named band of interest).

We followed the same procedure for the night playback birds. In this case, there was no need for a transformation of the outcome variable and no random factor was necessary. The results for each bird separately are shown in Supplementary Figure 8.

###### Day LFP analyses

**FIGURE 3:**
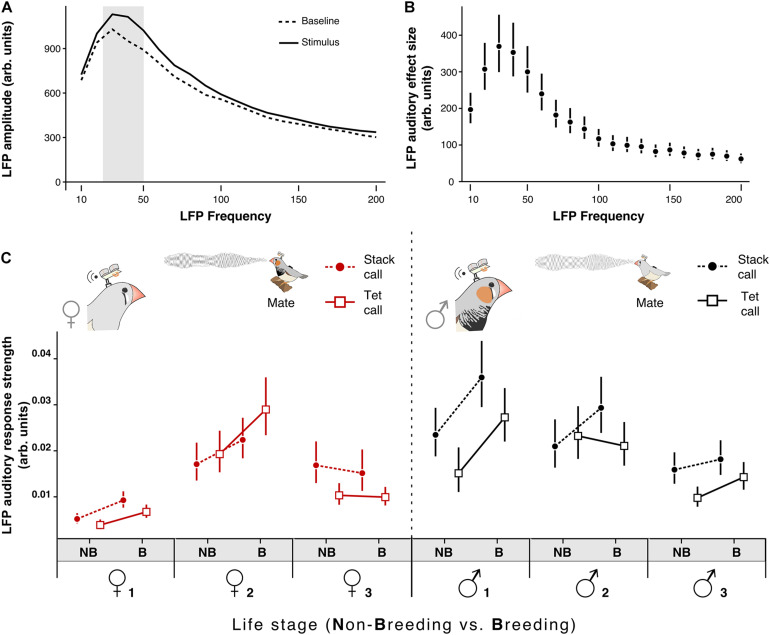
Onset of breeding conditions affects the NCM auditory responses. **(A)** Average LFP amplitude during Baseline and during the auditory Stimulus from a representative individual; the band of 30–50 Hz is the one with the highest amplitude modulation. **(B)** Average auditory amplitude modulation (Auditory-Stimulus) from a representative individual **(C)** Females’ (left) and males’ (right) LFP auditory auditory response strength (for the 30–50 Hz band) in response to the two most frequent contact vocalizations (stack call [circles and dotted line]; tet calls [squares and solid lines]) of their mate in the non-breeding (NB) and breeding (B) life stage. Circles and squares represent the estimated mean from the model and vertical bars the 95% CrI. A probability of difference higher than 95% can be assumed if the estimates of a group do not overlap the CrI of the other group.

After identifying the frequencies of maximal response toward the contact calls (30–50 Hz; Figure 3B) we performed three linear mixed effect models with this specific frequency band to estimate the change in the ARS after the breeding onset toward the different contact calls of the mate of focal males (*n* = 3) and females (*n* = 3) and also toward the single male calls. The outcome variable for the three models was the ARS of the maximal response bands transformed as: *Log(x^2^)*, where “*x*” is the ARS for each vocalization. For the first model, comparing the ARS toward tet and stack calls of the mate (Figure 3C), the explanatory variables were: treatment, call type, ID and their interactions. For the second and third model comparing the ARS of tet calls (Supplementary Figure 6) or stack calls (Supplementary Figure 7) from different emitters the explanatory variables were: treatment, emitter (Single Male vs. Mate), ID and their interactions. In the three models we included “day,” “call information” and “LFP frequency” as random factors to account for the repeated measures of the individuals across days, the different contexts of the acoustic stimuli (isolated, reply and replied) and the within frequency band variation (30, 40, and 50 Hz). For the exact fitted values and CrIs see Supplementary Table 6.

###### Night Playback LFP analyses

**FIGURE 4:**
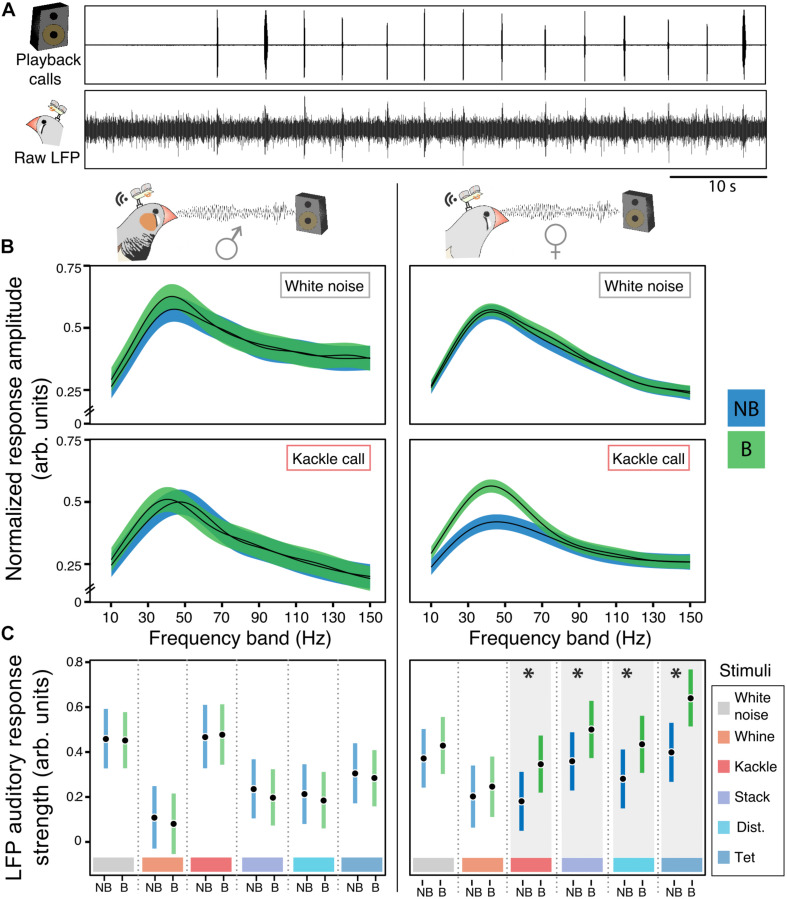
Females’ auditory responses toward night playbacks change selectively after the breeding onset. **(A)** Example of NCMs’ LFP responses toward playbacks. Raw LFP corresponds to the 300 Hz threshold low pass filtered response in NCM. **(B)** Males’ (left) and females’ (right) auditory LFP response amplitude in non-breeding (NB) and breeding **(B)** contexts in response to white noise and kackle breeding calls during playback. Black lines represent the mean and colored ribbons the 95% confidence intervals of the general additive mixed models. **(C)** Males’ (left) and females’ (right) estimated mean and 95% CrI for the NCM ARS in the 30–50 Hz band toward different playback stimuli in non-breeding (NB) and breeding **(B)** life stages. There is an effect (indicated by asterisks) of the breeding onset in females LFP response to kackle calls (posterior probability difference between NB and B = 99.83%), to stack calls (posterior probability difference between NB and B = 98.78%), distance calls (posterior probability difference between NB and B = 98.79%) and tet calls (posterior probability difference between NB and B higher than 99.99%) calls.

For illustrative purposes we performed general additive mixed models applying the function “*gamm*” of the package “*mgcv*” to visualize the LFP auditory response of males and females toward kackle calls and white noise (Figure 4B). Separate models were applied for males and females. The outcome variable was the mean root mean square of the different frequencies (10–150 Hz). The explanatory variables were treatment, call type and their interaction; being treatment: non-breeding (NB) and breeding (B), and call type: the different acoustic stimuli. We included “day” and “bird ID” as random factors, in this way we could account for the repeated measures and the between-individual variation. In Figure 4B we report the fit with the 95% confidence interval for the white noise and the kackle call only for illustration purpose and no conclusion is drawn from these results.

Next, we performed a linear mixed model for the frequency bands of interest to test the hypothesis that the breeding affects the LFP auditory response in NCM in these bands differentially according to the acoustic stimuli (Figure 4C; version with raw data points: Supplementary Figure 10). Both sexes were considered in the model (nmales = 3; nfemales = 3). The outcome variable for the three models was the mean ARS for the bands of interest. The explanatory variables were treatment, call type, sex and their interactions. This model included “bird ID,” “day” and “LFP frequency” to account for the individual variation, the repeated measures of the individuals across days and the within LFP frequency band variation. See Supplementary Table 7.

Additionally, we calculated one model for each bird (i.e., six models) to illustrate the effect of the breeding onset on the different calls from the mate at the individual level (Supplementary Figure 9). In these models the outcome variable was the ARS for the bands of interest and the explanatory variables were treatment, call type and their interaction. We also included “day” and “LFP frequency” to account for the repeated measures of the individuals across days and the within LFP frequency band variation. See Supplementary Table 10.

**FIGURE 5:**
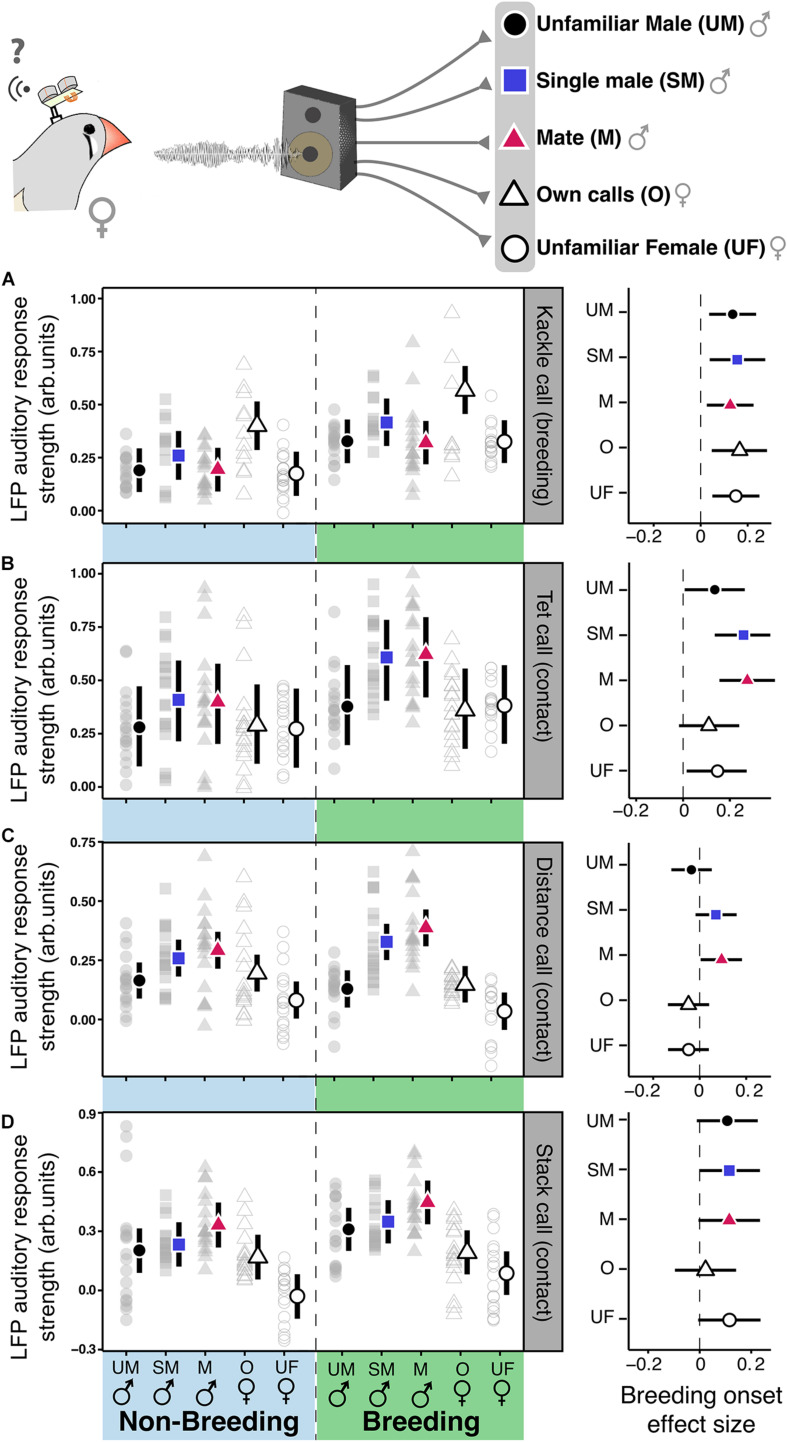
In females contact calls-related auditory plasticity in NCM depends on the identity of the emitter. Left panels show NCM’s auditory response strength in the low-gamma band toward breeding **(A)** and contact calls **(B–D)** of different emitters [Unfamiliar male [UM], Familiar Single Male (SM), Mate (M), Own calls (O), Unfamiliar Female (UF)] in non-breeding (NB) and breeding (B) contexts. Colored shapes represent the estimates, vertical bars the 95% CrI from the linear mixed models and gray shapes the raw data. The right panel of each segment **(A–D)** shows the model estimate and 95% CrI of the breeding induced effect size of the stimuli from different emitters within each call type. An statistically meaningful effect can be assumed if the effect sizes’ CrI do not overlap cero.

Finally, to test whether breeding induced auditory plasticity could be influenced by the identity of the emitter we performed linear mixed models for stack, tet, distance, and kackle calls of different familiarity for the females (Figures 5A–D). We performed one model for each call type and the outcome variable was LFP ARS for the bands of interest. The explanatory variables were treatment (non-breeding and breeding) and call origin (mate male, familiar single male, and unfamiliar male). As for the previous playback models we included “ID” and “LFP frequency” as random factors. We determined the response to be statistically different between treatments if the posterior probability of difference was larger than 95%. See Supplementary Table 9.

## Results

### Effect of Breeding Onset on Vocal Behavior and Estrogen Levels

To assess the change in vocal dynamics between mates we first recorded a trio (a mated pair group-living with an additional male, named single male) in non-breeding context via wireless individual microphones (Figure 1D). After this, we experimentally induced the breeding onset by adding variety of food, nesting sites, plants (Figure 1D), and continued recording the birds’ vocal interactions. Then we analyzed call-based vocal communication within pairs for the entire repertoire. For the different combinations of call interactions we calculated two parameters that describe vocal dynamics: reply strength (RS) and the reply latency (RL) (see section “Materials and Methods”). After the introduction of nesting material all individuals within a pair (except for one male) increased the number of breeding call emissions (Figure 2A, posterior probability of increase higher than 99.9%, Supplementary Table 1) but only females increased their circulating estrogen levels (Figure 2B; posterior probability of increase of 99.86%; *Estimate [95% CrI] for Non-breeding* = *40.35 [25.3; 63.73] pg/ml; for Breeding* = *85.62 [53.25; 137.99] pg/ml*; Supplementary Table 2). Regarding the vocal interactions, both sexes increased the RS and decreased the RL toward their partner’s breeding calls after the environmental enrichment (i.e., elicited more breeding calls as replies to the partner compared to the baseline and did so faster, Figure 2C and Supplementary Table 3) but only females decreased their RL toward male contact call vocalizations (e.g., “Tet-Stack” and “Distance” in Figure 2C and Supplementary Table 3). Overall, both sexes reacted to the breeding onset and modified the RS and RL toward each other’s breeding calls, but only females reacted to it by increasing their estrogen levels and changing the timing (i.e., respond faster) of their replies to their mates’ contact calls.

### Effect of Breeding Onset on NCM Responses During Vocal Interactions

To test whether behavioral changes in vocal dynamics co-occurred with changes in auditory responses in a secondary auditory area we repeated the experiment studying the vocal transition from non-breeding to breeding context while implanting the focal couple with miniature electrophysiological wireless transmitters to record the neural activity of the NCM (Schregardus et al., 2006; Ter Maat et al., 2014; Figure 1C; see section “Materials and Methods”). Implanted birds behaved normally, e.g., engaged in nest building (Supplementary Video 1). After the breeding onset NCM-Implanted birds increased the rate of breeding call emission similarly to non-implanted birds (Supplementary Figure 1 and Supplementary Table 1) as well as their vocal interactions (Supplementary Figures 2, 3 and Supplementary Table 3). Further, NCM-implanted females increased their circulating estrogen levels (Supplementary Figure 4 and Supplementary Table 2). We found that the LFP frequency band with the maximal amplitude in response to the acoustic stimuli both in males and females was between 30 and 50 Hz in the low-gamma band (Figures 3A,B; data for each individual shown in Supplementary Figure 5 and Supplementary Table 4, see section “Materials and Methods”). In five (the three males and two females) out of six birds the breeding onset induced an increase in the LFP auditory response strength (ARS, see section “Materials and Methods”) in response to at least one of the two most common contact calls of their mate: stack and tet calls (Figure 2C; Posterior probability of increase higher than 99.9%; Supplementary Table 5). When comparing the ARS of the mate calls with that of the single male calls we found no difference for the tet calls (Supplementary Figure 6 and Supplementary Table 6). However, independent of life stage, in four (the three males and one female) out of six birds the LFP ARS toward the stack calls of the mate was higher than the one toward the stack calls of the single male (Supplementary Figure 7; Posterior probability of difference higher than 99.2%; Supplementary Table 6). Birds did not produce a large enough number of breeding and distance calls in the non-breeding context to allow a comparison of related LFPs between contexts.

### Effect of Breeding on the NCM Response to Different Vocalizations During Night Playbacks

During daytime interactions, not only the acoustic soundscape is complex but also additional mechanisms beyond those in the auditory system *per se* could influence our measure of auditory response, such as individual attentiveness or expectation for instance (Fritz et al., 2007). Hence, we decided to confirm and extend the findings on auditory plasticity during the day by investigating auditory responses to nighttime playbacks (Figure 4A). In such a controlled acoustic environment, we could investigate breeding induced changes that were specific to the auditory system by minimizing the interaction with other sensory inputs. During 2 days before and 2 days after the breeding onset we presented the entire repertoire of each bird’s partner plus an internal control, white noise (see section “Materials and Methods”). As during daytime interactions, during playbacks the maximum ARS to the sounds played was also in the frequency band between 30 and 50 Hz (Supplementary Figure 8). After breeding onset, females increased their low-gamma ARS in response to three types of contact and one breeding call (Figures 4B,C posterior probability of increase higher than 95%, Supplementary Table 7). Breeding onset had no effect on white noise internal control, whine breeding calls or song (Figure 4C and Supplementary Tables 7, 8 for the song). In contrast to females, there was no change in the males’ LFP responses toward any stimulus during the night playbacks (Figure 4C).

### Effect of Breeding Onset on Females’ NCM Response to Calls From Different Emitters

To investigate whether context-induced auditory plasticity in females depended on the identity of the emitter we played back contact and breeding calls of (1) the single male, (2) an unfamiliar male, (3) the females’ own calls, and (4) those of an unfamiliar female. For the kackle breeding calls, the breeding onset caused an increase of the LFP ARS irrespective of the emitter identity. The breeding onset also increased the ARS for the birds’ own call (Figure 5A, posterior probability of an increase higher than 99.3% for the kackles of every emitter; Supplementary Table 9). Intriguingly, compared to the kackles of the other emitters the birds own kackle call elicited the highest ARS independent of the context (Figure 5A, left panel, posterior probability higher than 99.2% vs. the kackle of every emitter; Supplementary Table 9). On the contrary, for tet and distance contact calls, the effect of breeding onset was emitter-specific. Only the ARS toward the calls of the mate and the single male increased in the breeding context (Figures 5B,C, posterior probability of an increase: Distance call of the mate, 98.47%; Distance call of the single male, 94.04%; Tet call of the mate, 99.5%; Tet call of the single male, 99.73%; Supplementary Table 9). Further, after the breeding onset tet and distance calls from familiar birds (i.e., the mate and the single male) produced a higher ARS than that of the other emitters (Figures 5B,C, left panel, posterior probability of a difference higher than 99.5% vs. the calls of unfamiliar emitters; Supplementary Table 9). For stack contact calls the breeding onset influenced the ARS to every stimulus except to the females’ own call (Figure 5D, right panel, posterior probability higher than 99% for every emitter except the birds own stack call; Supplementary Table 9). Furthermore, after the breeding onset male stack calls elicited a higher ARS than females’ and within the males, the call of the mate had the highest ARS (Figure 5D; posterior probability higher than 95%; Supplementary Table 9). Thus, the effect of the context modulated the auditory systems’ response in an emitter-specific manner toward different call types.

## Discussion

We demonstrated breeding context-induced auditory plasticity in the receiver brain in freely interacting animals. Through playback experiments we found that auditory plasticity was long lasting only in females and, because it encompassed only specific vocal categories, it did not reflect an overall change in the auditory system. Specifically, the call type, the identity of the sender and the context interact to determine the plasticity of a high-order auditory region.

In our study, the bands between 30 and 50 Hz of the LFP low-gamma band showed the strongest ARS toward acoustic stimuli. LFPs in the gamma-band are associated with multiple behaviors (Ni et al., 2016) including vocalizations (Lakatos et al., 2005). In anesthetized zebra finches the power of gamma activity correlates with local firing rates within approximately 200 μm of the electrode tip in auditory regions (Schachter, 2016). Therefore, we are confident that the measured responses arose from NCM. In addition, because of NCMs sparse coding properties (Kozlov and Gentner, 2016), it is likely that the observed activity also reflects the response of neural networks and not only a local effect within the vicinity of the electrode.

When comparing day with night LFP, the ARS during night playback was higher than that during the day. Most likely, this is because of the differences in the soundscape between conditions (freely-behaving interactions vs. playbacks) that influenced the normalization of the response strength of the LFP recordings (see section “Materials and Methods”) and not necessarily a difference in NCMs’ responsiveness between contexts. Specifically, the absence of noise and movement during night playback probably allowed for clean high signal-to-noise ratio auditory recordings, whereas during active interactions the presence of different biotic and abiotic factors (e.g., wing flap noise and movement) increased the noise level. For these reasons, although there can be processing differences between day and night in NCM (Yanagihara and Yazaki-Sugiyama, 2016), we limit ourselves to a discussion of the results within each context separately.

We found breeding induced auditory plasticity in interacting birds and during night playbacks. However, in interacting birds auditory plasticity was not sex specific during the day, and during nighttime playbacks only females showed changed responses depending on the breeding situation. Further, only females increased their circulating estrogen levels after the breeding onset. Thus, it is possible that different mechanisms are taking place between sexes. For example, during interactions, males could enhance their auditory responses during breeding by changing their attentiveness or expectation toward the emitter’s vocalizations (Fritz et al., 2007). Alternatively, females might be experiencing a long lasting breeding-induced change in their auditory system mediated by estrogens (Arch and Narins, 2009) due to their systemic and sustained estrogenic response to breeding onset. This could explain why auditory changes persisted during the night in females and not in males.

We observed changes in females’ auditory LFP toward specific vocalizations during playback. These signal specific changes could be linked to an adaptive change in saliency of signals with particular relevance during breeding. Zebra finches are highly social, and have a diverse repertoire (Zann, 1996) whose composition changes during the life cycle as shown in the current study. While the song has a role in mate attraction and male-male competition (Gil and Gahr, 2002), innate calls have different functions at the pair level as for example coordinating reproductive (Boucaud et al., 2017) or other pair-related behaviors (Gill et al., 2015; D’Amelio et al., 2017b). Thus, signal-specific life-stage dependent auditory plasticity could be a mechanism for changing the saliency of specific vocal signals during the breeding period.

We found that in females auditory LFPs toward kackle breeding calls increased with breeding onset irrespective of the identity of the emitter during the playbacks. Here, NCM exhibited a unique response toward the birds’ own call. Only in this call category there was a breeding-induced increase of the ARS toward the bird’s own vocalization and it was also the only case where the ARS toward the birds’ own call was the strongest compared with that of other emitters. This could be caused by self-stimulation, which has been thoroughly studied in the innate cooing behavior of ring doves (*Streptopelia roseogrisea*) [reviewed by Cheng (1992)]. During cooing interactions upon breeding, female ring doves respond physiologically to their own vocalizations that are triggered by the male vocalizations at the nest (Cheng, 1992) with estrogen involved in the physiological response (Gibson and Cheng, 1979). Zebra finches also exchange breeding calls in a coordinated manner in a comparable way to that of ring doves (Miller and Miller, 1958; Boucaud et al., 2017). Thus, self-stimulation could be taking place in zebra finches too.

Females changed their ARS toward contact calls (tet and distance calls) from familiar birds only (i.e., the mate and the single male). This provides the first evidence for an emitter-specific auditory change induced by a biologically meaningful environmental change. Contact calls are important for pair bond formation (Gill et al., 2015; D’Amelio et al., 2017b) and carry information about individual identity (D’Amelio et al., 2017a; Elie and Theunissen, 2018). In line with the social nature of zebra finches and their mating system (Zann, 1996), the strength of the response toward familiar individuals suggests that responsiveness to contact calls of known group members is specifically enhanced during the reproductive period. This enhanced response could be adaptive for strengthening the attention toward individuals that are familiar to the birds (i.e., mate and group members) during a critical life-history stage such as reproduction.

Given the duration of this study we could not measure single or multi-unit spiking activity together with the LFP. Thus, we cannot make strong inference about the cellular mechanisms that explain our results from an electrophysiological perspective. A plausible physiological mechanism for the observed sex-specific modulation of the response amplitude is the activity of estrogen. Estrogen is known to affect the vertebrate auditory system [reviewed by Caras (2013)] and there is evidence from pharmacological experiments for its role as a neuromodulator in the NCM [reviewed in Pinaud and Tremere (2012)]. Estrogen augments NCM neurons’ firing rate and selectivity (Remage-Healey et al., 2010; Remage-Healey and Joshi, 2012). Although songbirds can produce estrogen from androgenic precursors in the NCM (Schlinger and Arnold, 1992), it is likely that the sex-specific auditory change that we observe during nighttime is related to the systemic increase of estrogen that occurs in females after onset of breeding activities. Given that males can also generate estrogen from testosterone *via* the enzyme aromatase in the NCM (Schlinger and Arnold, 1992) the absence of a life-stage dependent effect in males during the night might seem surprising. However, there is good evidence for fine scale sexual differences in the local synthesis of estrogen within NCM. For example males and females differ in the number of aromatase-positive synapses (Peterson et al., 2005) as well as in their neural organization (Krentzel et al., 2018). In this way, males and females cannot only differ regarding the timing and context where estrogen acts, but also in where within the NCM estrogen is locally elevated. Nevertheless, this aromatase-mediated mechanism appears linked to fast events (e.g., social interactions) (Remage-Healey et al., 2008) that modulate auditory processing in an acute manner (Remage-Healey, 2012). Although this could play a role during the daytime interactions, it is unlikely that this mediates a phenomenon with a long temporal scale such as life-stage dependent auditory plasticity. Although the underlying mechanism is speculative, our results demonstrate a sexually distinct and long-lasting response to the breeding onset of NCM of zebra finches.

In females, life-stage dependent auditory plasticity was signal-specific. After the breeding onset, LFP responses increased only to specific contact and breeding calls. The LFP did not increase in response to song or the white noise control, for example. As in fish and anurans (Sisneros et al., 2004; Arch and Narins, 2009), estrogen can affect the auditory system at the peripheral level in birds (Noirot et al., 2009). With such a mechanism we would have expected general, and not stimulus- or emitter-specific auditory changes, since all calls cover wide frequency spectra (see Figure 1A). Zebra finches can discriminate and recognize individuals from acoustic signatures for most of the vocalizations of their repertoire, even though they are innate (D’Amelio et al., 2017a; Elie and Theunissen, 2018). Thus, it is likely that the signal specificity of auditory plasticity that we describe in females is a consequence of estrogenic actions on specific neural circuits linked to the semantic categories encoded in the NCM (Elie and Theunissen, 2015). The combination of NCMs’ distributed and sparse coding properties (Elie and Theunissen, 2015; Kozlov and Gentner, 2016) with the scattered distribution of estrogen receptors in NCM (Gahr et al., 1993) provide a reasonable scenario for such a circuit-specific mechanism to take place.

## Conclusion

We provide evidence for a tight interaction between vocalization type and auditory plasticity, a mechanism that can change the saliency of vocal signals across contexts. By conducting long-term neural and vocal recordings of freely interacting birds while mimicking a natural environmental change in combination with playback experiments, we discovered that an area analogous to the mammalian auditory association cortex changes its neural responsiveness only toward certain functionally distinct vocalizations and from specific emitters. Altogether, our results significantly widen our understanding of the interaction between sensory plasticity and signal categories, two elements that are pivotal for vocal communication evolution.

## Data Availability Statement

The raw data supporting the conclusions of this article will be made available by the authors, without undue reservation.

## Ethics Statement

All housing and experimental procedures were approved by the Government of Upper Bavaria (ROB-55.2-2532.Vet_02-19-127).

## Author Contributions

NA conceived and designed the study, performed the experiments, analyzed the results, and wrote the manuscript with input from all authors. PD’A provided advice on the study design. PD’A, AM, and MG provided the advice on the interpretation of the results. All authors contributed to the article and approved the submitted version.

## Conflict of Interest

The authors declare that the research was conducted in the absence of any commercial or financial relationships that could be construed as a potential conflict of interest.
