# Supplementary material for: Life-Stage Dependent Plasticity in the Auditory System of a Songbird Is Signal and Emitter-Specific
Source: Front Neurosci. 2020 Dec 4;14:588672. doi: 10.3389/fnins.2020.588672 (PMC7746620; doi:10.3389/fnins.2020.588672)
Supplement: Supplementary file 1 [file Table_1.DOCX]

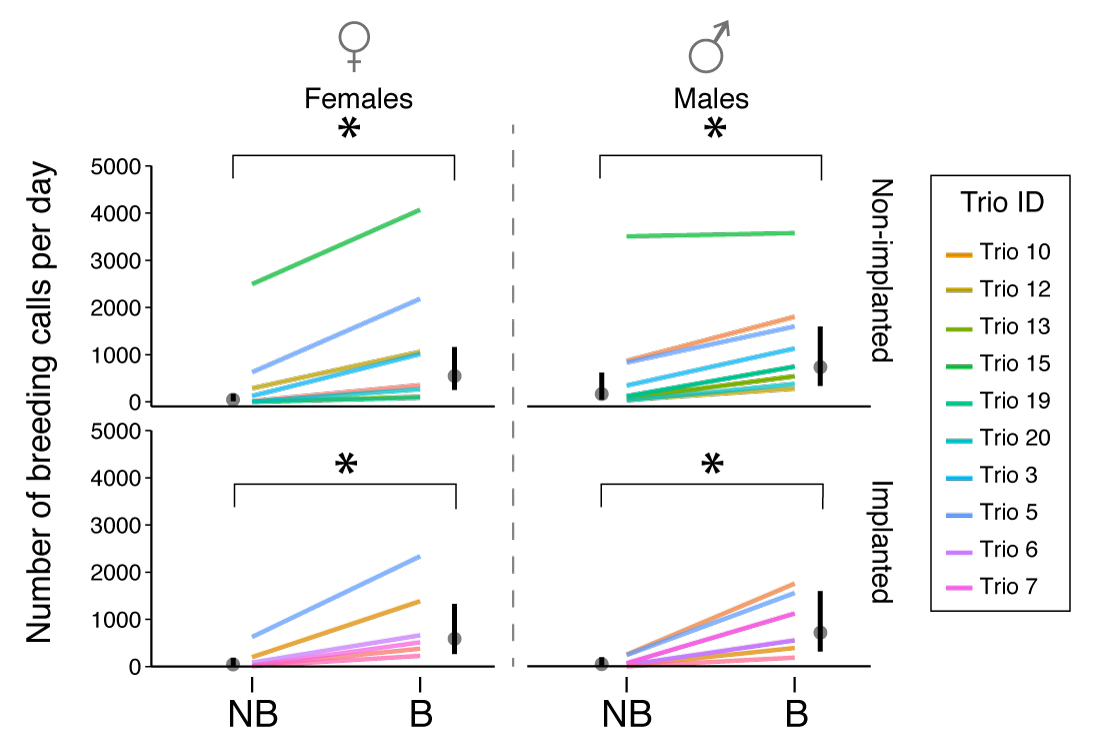


## Figure S1 | Number of breeding calls in non-breeding (NB) and breeding (B) condition in implanted and non-implanted males and females. Black shapes represent estimates from the model, vertical lines represent 95% CrI and each line the change of one individual. Asterisks represent a posterior probability of difference higher than 95%.

| \|  \| **Log (Breeding calls per day)** \| \| --- \| --- \| \| ***Fixed effects β (95% CrI)*** \| \| \| Intercept \| 3.81 (2.49; 5.12) \| \| Treatment \| 2.50 (1.59; 3.43) \| \| Trial (NCM-Implant) \| -0.002 (-1.18; 1.17) \| \| Sex (Male) \| 1.30 (-0.55; 3.18) \| \| Treatment * Trial \| 0.07 (-1.04; 1.20) \| \| Treatment * Sex \| - 1.01 (-2.30; 0.31) \| \| Trial * Sex \| -1.22 (-2.86; 0.44) \| \| Treatment * Tria * Sex \| 1.12 (-0.47; 2.67) \| \| ***Random factors σ^2^ (95% CrI)*** \| \| \| ID (Slope) \| 1.81 (1.52, 2.02) \| \| ID (Intercept) \| 5.33 (3.46; 7.84) \| |
| --- | --- | --- | --- | --- | --- | --- | --- | --- | --- | --- | --- | --- | --- | --- | --- | --- | --- | --- | --- | --- | --- | --- | --- | --- | --- | --- |

## Table S1 | Effect of the breeding onset (Treatment) on the number of breeding calls of males (Mate) and females for two set of experiments, with brain implant and without (Behaviour).

Generalized linear mixed effect model:

Log(Number of calls)~Treatment*Trial*Sex + (ID|Treatment)

|  | **Non-Implanted birds Log (E2) pg/ml** | **NCM-Implanted birds Log (E2) pg/ml** |
| --- | --- | --- |
| **Fixed effects β (95% CrI)** | | |
| **Intercept (NB, Female)** | 3.702 (3.245; 4.156) | 3.676 (3.139; 4.233) |
| **B1** | 0.408 (-0.351; 1.148) | 0.755 (0.050; 1.462) |
| **B2** | 0.752 (0.143; 1.380) | 1.021 (0.323; 1.715) |
| **Sex** | -0.669 (-1.296; -0.015) | -0.540 (-1.252; 0.176) |
| **B1*Sex** | -0.583 (-1.645; 0.489) | -0.288 (-1.300; 0.679) |
| **B2*Sex** | -0.537 (-1.417; 0.332) | -0.711 (-1.710; 0.270) |
| **Random factors σ^2^ (95% CrI)** | | |
| **Experimental Unit (*trio*)** | 0.04 (0.016; 0.089) | 0.107 (0.028; 0.286) |
| **ID** | 0.00 (0.00;0.00) | 0.00 (0.00;0.00) |

## Table S2 | Effect of the breeding onset (Treatment) on the estrogen (E2) levels of males and females for two set of experiments, non-implanted birds and NCM-implanted birds.

Generalized linear mixed effect model for each set:

Log(E2)~Treatment *Sex + (1/Treatment|ID)


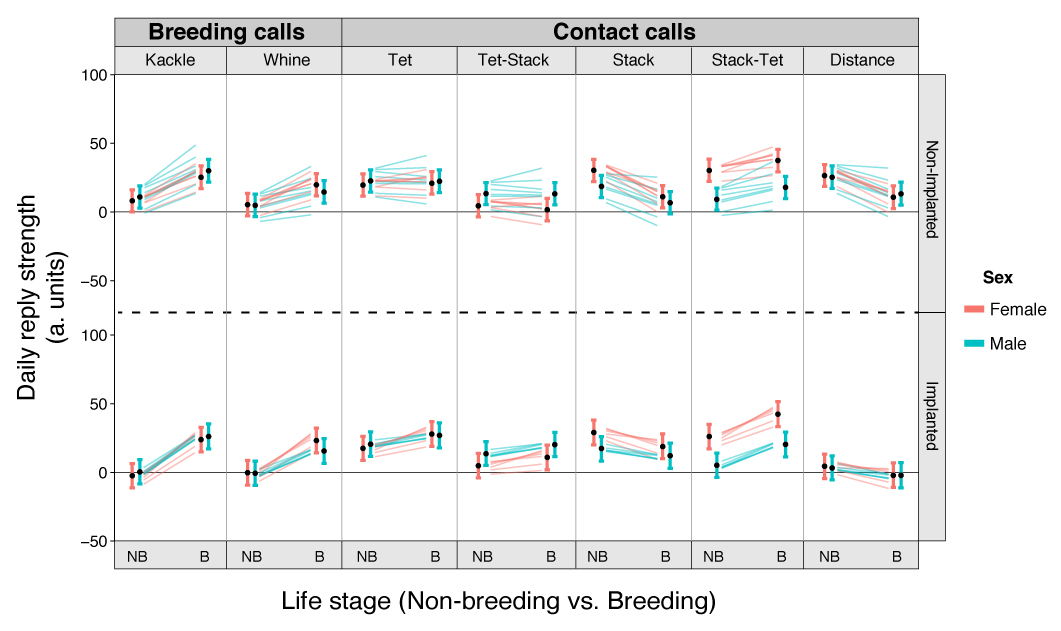


## Figure S2 | Change in the reply strength of non-implanted (top) and implanted (bottom) males (blue) and females (red) towards each other with the most common call interactions. The call names represent an interaction with that specific call (For example, ‘tet’ refers to the response strength of implanted and non-implanted males and females towards each other using this call type. ‘tet-stack’ and ‘stack-tet’ are asymmetric ‘female-male’ vocal interactions). Black shapes represent estimates from the model, vertical lines represent 95% CrI and each line the average change for an individual.


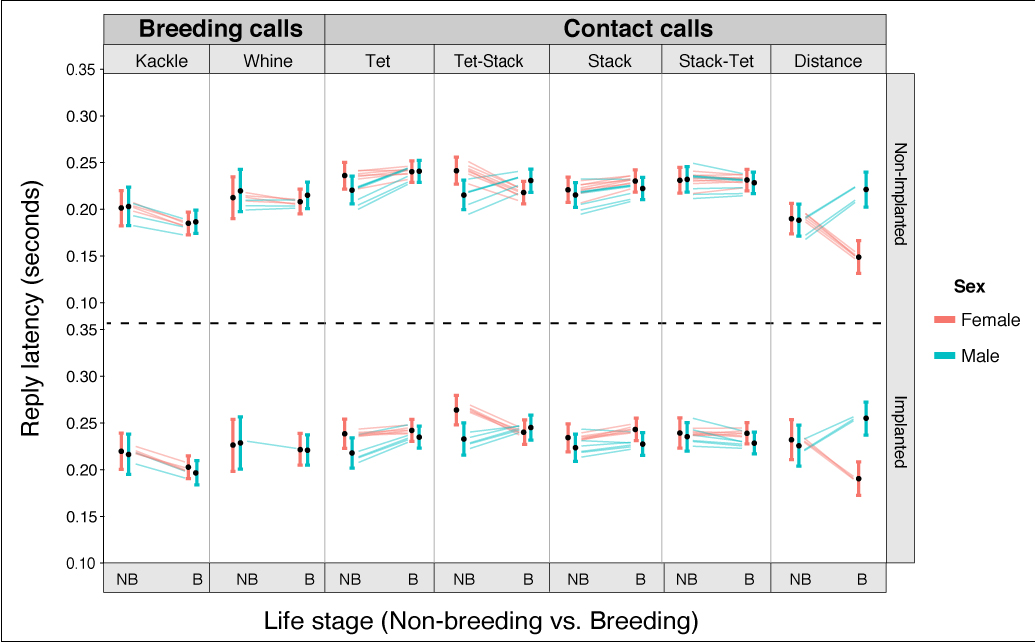


## Figure S3 | Change in the reply latency of non-implanted (top) and implanted (bottom) males (blue) and females (red) towards each other with the most common call interactions. The call names represent an interaction with that specific call (For example, ‘tet’ refers to the response strength of implanted and non-implanted males and females towards each other using this call type. ‘tet-stack’ and ‘stack-tet’ are asymmetric ‘female-male’ vocal interactions). Black shapes represent estimates from the model, vertical lines represent 95% CrI and each line the average change for an individual.

|  | **Reply Strength**  **(RS-arb. units)** | **Reply Latency**  **(RL - sec)** |
| --- | --- | --- |
|  |  |  |
| **Fixed effects β (95% CrI)** | | |
| **Intercept (NB; Kackle Call; Female; Non-Implanted)** | 8.08 (0.11; 16.20) | 0.202 (0.183; 0.220) |
| **Treatment (B)** | 17.21 (7.44; 26.80) | -0.017 (-0.036; 0.003) |
| **Trial (NCM-Implant)** | 0.04 (-0.61; 0.71) | 0.018 (0.001; 0.035) |
| **Sex (Male)** | 2.87 (-8.19; 13.60) | 0.002 (-0.026; 0.029) |
| **Call Type - Whine** | -2.75 (-12.49; 6.95) | 0.011 (-0.016; 0.037) |
| **Call Type - Tet** | 11.43 (1.57;20.94) | 0.034 (0.014;0.055) |
| **Call Type - Tet-Stack** | -3.52 (-13.50; 6.18) | 0.040 (0.020; 0.061) |
| **Call Type - Stack** | 22.13 (12.32; 31.77) | 0.019 (-0.001; 0.039) |
| **Call Type - Stack-Tet** | 22.23 (12.46; 31.76) | 0.030 (0.01; 0.049) |
| **Call Type - Distance** | 18.34 (8.52; 27.94) | -0.011 (-0.034; 0.011) |
| **Treatment (B) * Trial (NCM-Implant)** | 9.06 (1.67; 16.78) | 0.000 (-0.014; 0.013) |
| **Treatment (B) * Sex (Male)** | 1.89 (-11.54; 15.35) | 0.000 (-0.029; 0.029) |
| **Trial (NCM-Implant) * Sex (Male)** | -0.01 (-8.97; 9.15) | -0.005 (-0.023; 0.013) |
| **Treatment (B) * Whine** | -2.73 (-14.76; 9.21) | 0.012 (-0.018; 0.042) |
| **Treatment (B) * Tet** | -15.75 (-27.93; -3.69) | 0.021 (-0.002; 0.044) |
| **Treatment (B) * Tet-Stack** | -20.18 (-31.99; -7.78) | -0.007 (-0.030; 0.017) |
| **Treatment (B) * Stack** | -36.33 (-48.49; -24.31) | 0.026 (0.003;0.048) |
| **Treatment (B) * Stack-Tet** | -10.06 (-22.07; 1.78) | 0.017 (-0.006; 0.040) |
| **Treatment (B) * Distance** | -32.89 (-44.92; -20.90) | -0.024 (-0.053; 0.003) |
| **Sex (Male) * Whine** | -3.25(-16.02; 9.37) | 0.006 (-0.032; 0.043) |
| **Sex (Male) * Tet** | 0.26 (-12.61; 12.92) | -0.017 (-0.031; -0.001) |
| **Sex (Male) * Tet-Stack** | 5.97 (-6.81; 18.56) | -0.028 (-0.057; 0.001) |
| **Sex (Male) * Stack** | -14.52 (-27.54; -1.76) | -0.007 (-0.036; 0.021) |
| **Sex (Male) * Stack-Tet** | -23.90 (-36.56; -11.20) | -0.001 (-0.029; 0.027) |
| **Sex (Male) * Distance** | -4.02 (-17.00; 8.88) | -0.003 (-0.035; 0.029) |
| **Trial (NCM-Implant) * Whine** | 4.90 (-3.62; 13.56) | -0.004 (-0.024; 0.015) |
| **Trial (NCM-Implant) * Tet** | 8.52 (-0.23; 17.32) | -0.016 (-0.031; -0.001) |
| **Trial (NCM-Implant) * Tet-Stack** | 10.78 (2.07; 19.64) | 0.004 (-0.011; 0.020) |
| **Trial (NCM-Implant) * Stack** | 9.35 (0.62; 18.14) | -0.005 (-0.020; 0.010) |
| **Trial (NCM-Implant) * Stack-Tet** | 6.40 (-2.25; 14.98) | -0.010 (-0.025; 0.005) |
| **Trial (NCM-Implant) * Distance** | -11.42 (-20.03; -2.58) | 0.024 (0.004; 0.044) |
| **Treatment (B) * Trial (NCM-Implant) * Sex (Male)** | -2.44 (-13.34; 8.17) | -0.003 (-0.022; 0.016) |
| **Treatment (B) * Sex (Male) * Whine** | -6.82 (-24.11; 10.29) | 0.000 (-0.043; 0.043) |
| **Treatment (B) * Sex (Male) * Tet** | -3.64 (-20.80; 13.21) | 0.016 (-0.018; 0.050) |
| **Treatment (B) * Sex (Male) * Tet-Stack** | 0.91 (-19.39; 18.12) | 0.039 (0.005; 0.074) |
| **Treatment (B) * Sex (Male) * Stack** | 5.30 (-11.86; 22.61) | -0.002 (-0.036; 0.031) |
| **Treatment (B) * Sex (Male)* Stack-Tet** | -0.44 (-17.74; 16.54) | -0.004 (-0.037; 0.030) |
| **Treatment (B) * Sex (Male) * Distance** | 1.80 (-15.43; 18.91) | 0.074 (0.034; 0.115) |
| **Random factors σ2 (95% CrI)** | | |
| **ID (Slope)** | 11.31 (8.32; 12.72) | 0.00009 (0.00008; 0.0001) |
| **ID (Intercept)** | 35.64 (23.58; 51.32) | 0.0002 (0.0001; 0.0003) |

## Table S3 | Effect of the breeding onset (Treatment) on the reply strength (RS) and reply latency (RL) of non-implanted and NCM-implanted males and females using different call type exchanges.

##

## Figure S4 | Estrogen levels of non-implanted (A) and NCM-Implanted (B) males (Right panel, Back CrI) and Females (Left panel, Red CrI) in non-breeding condition (NB-first 24 hs of experiment) and breeding condition (B1 24 hs after breeding onset, and B2 24 hs after the last day of experiment). Black shapes represent estimates from the model, vertical lines represent 95% CrI and grey shapes the raw values for every bird.


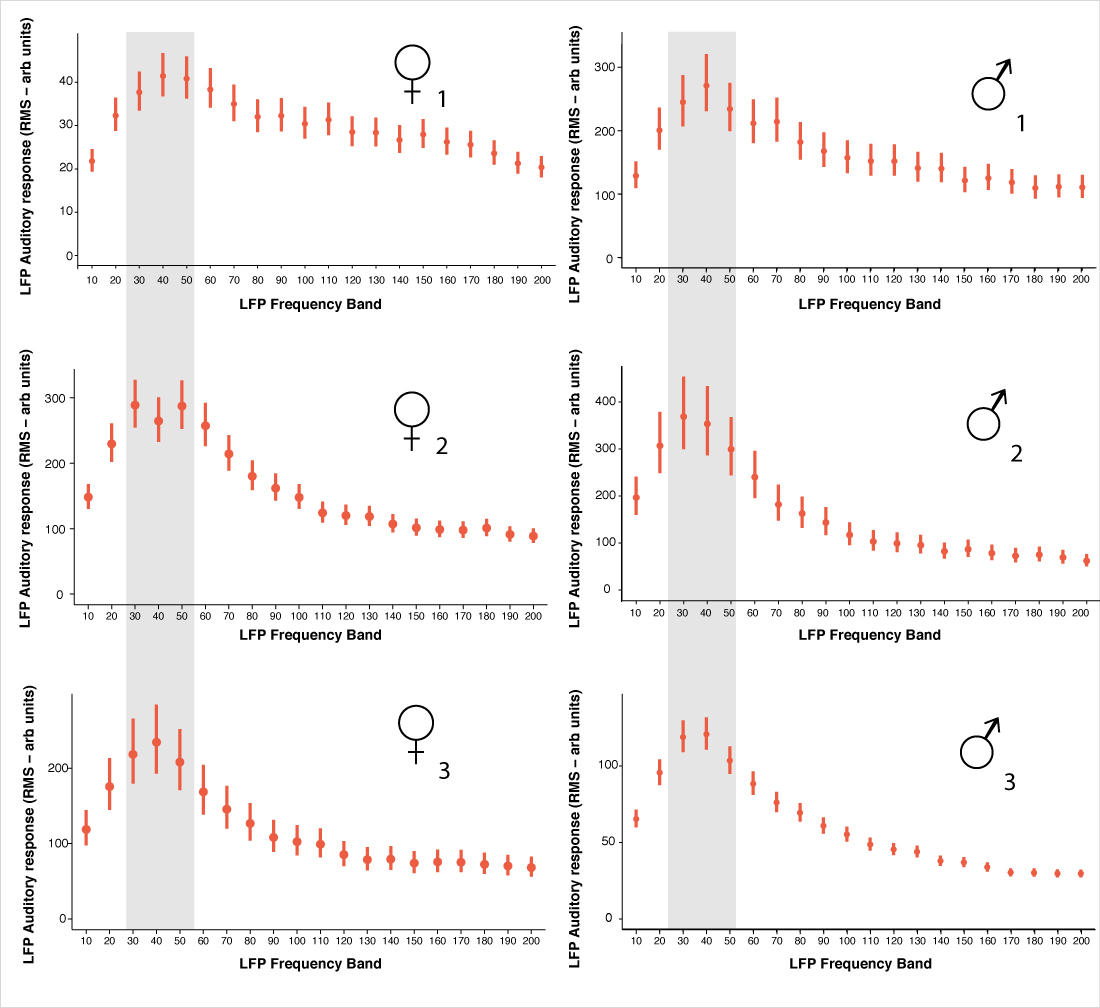


## Figure S5 | Average auditory amplitude modulation (Auditory-Baseline) from each individual during daily interactions, the band of 30-50 Hz (marked grey) is the one with the highest amplitude modulation.

|  | **Female 1 Log(Auditory Effect Size^2)** | **Female 2 Log(Auditory Effect Size^2)** | **Female 3 Log(Auditory Effect Size^2)** | **Male 1 Log(Auditory Effect Size^2)** | **Male 2 Log(Auditory Effect Size^2)** | **Male 3 Log(Auditory Effect Size^2)** |
| --- | --- | --- | --- | --- | --- | --- |
|  |  |  |  |  |  |  |
| ***Fixed effects β (95% CrI)*** | | | | | | |
| **Intercept (10 Hz Band)** | 6.16 (5.92; 6.40) | 9.67 (9.38; 9.95) | 8.78 (8.59; 8.98) | 9.72 (9.39; 10.04) | 10.57 (10.15; 10.97) | 8.36 (8.18; 8.54) |
| **20 Hz** | 0.79 (0.55; 1.03) | 0.83 (0.52; 1.14) | 0.97 (0.74; 1.20) | 0.89 (0.52; 1.27) | 0.89 (0.52; 1.25) | 0.76 (0.52; 1.00) |
| **30 Hz** | 1.10 (0.85; 1.34) | 1.32 (1.01; 1.63) | 1.46 ( 1.23; 1.69) | 1.27 (0.89; 1.65) | 1.26 (0.90; 1.63) | 1.20 (0.96; 1.43) |
| **40 Hz** | 1.29 (1.04; 1.53) | 1.23 (0.91; 1.54) | 1.35 ( 1.12; 1.58) | 1.49(1.10; 1.88) | 1.17 (0.80; 1.53) | 1.23 (0.99; 1.47) |
| **50 Hz** | 1.26 (1.02; 1.50) | 1.49 (1.18; 1.80) | 1.01 (0.77; 1.24) | 1.20(0.82; 1.58) | 0.84 (0.47; 1.21) | 0.92 (0.68; 1.16) |
| **60 Hz** | 1.13 (0.89; 1.37) | 1.24 (0.93; 1.55) | 0.66 (0.43; 0.89 | 0.99 (0.62; 1.37) | 0.40 (0.39; 0.76) | 0.60 (0.37; 0.84) |
| **70 Hz** | 0.95 (0.71; 1.19) | 0.80 (0.50; 1.11) | 0.41 (0.18; 0.64) | 1.02 (0.64; 1.37) | -0.15 (-0.51; 0.24) | 0.31 ( 0.07; 0.54) |
| **80 Hz** | 0.77 (0.53; 1.01) | 0.59 (0.28; 0.90) | 0.16 (-0.07; 0.49) | 0.69 (0.32; 1.08) | -0.38 (-0.74; -0.01) | 0.12 (-0.12; 0.35) |
| **90 Hz** | 0.79 (0.54; 1.03) | 0.29 (-0.03; 0.60) | -0.15 (-0.38; 0.09) | 0.53 (0.15; 0.90) | -0.63 (-0.99; -0.26) | -0.14 (-0.38; 0.10) |
| **100 Hz** | 0.67 (0.42; 0.91) | -0.01 (-0.32; 0.31) | -0.35 (-0.58; -0.12) | 0.40 (0.02; 0.79) | -1.04 (-1.40; -0.67) | -0.33 (-0.58; -0.10) |
| **110 Hz** | 0.73 (0.48; 0.97) | -0.10 (-0.41; 0.21) | -0.39 (-0.62; -0.16) | 0.33 (-0.04; 0.71) | -1.29 (-1.65; -0.92) | -0.59 (-0.83; -0.35) |
| **120 Hz** | 0.54 (0.29; 0.78) | -0.33 (-0.64; -0.020) | -0.44 (-0.67; -0.21) | 0.33 (-0.04; 0.70) | -1.37 (-1.72; -1.00) | -0.73 (-0.97; -0.49) |
| **130 Hz** | 0.53 (0.29; 0.77) | -0.43 (-0.73; -0.12) | -0.44 (-0.67; -0.21) | 0.18 (-0.20; 0.56) | -1.45 (-1.81; -1.09) | -0.79 (-1.03; -0.56) |
| **140 Hz** | 0.40 (0.16; 0.65) | -0.44 (-0.76; -0.13) | -0.60 (-0.83; -0.37) | 0.17 (-0.21; 0.56) | -1.75 (-2.11; -1.28) | -1.09 (-1.33; -0.86) |
| **150 Hz** | 0.50 (0.25; 0.74) | -0.60 (-0.91; -0.30) | -0.57 (-0.80; -0.33) | -0.12 (-0.49;0.26) | -1.64 (-2.00; -1.28) | -1.14 (-1.38; -0.89) |
| **160 Hz** | 0.37 (0.13; 0.61) | -0.70 (-1.01; -0.39) | -0.53 (-0.77; -0.29) | -0.05 (-0.43; 0.32) | -1.85 (-2.21; -1.48) | -1.31 (-1.56; -1.07) |
| **170 Hz** | 0.32 (0.08; 0.57) | -0.70 (-1.01; -0.39) | -0.52 (-0.75; -0.29) | -0.17 (-0.53; 0.21) | -1.99 (-2.35; -1.62) | -1.53 (-1.77; -1.30) |
| **180 Hz** | 0.16 (-0.09; 0.40) | -0.66 (-0.97; -0.36) | -0.70 (-0.93; -0.47) | -0.32 (-0.70; 0.05) | -1.93 (-2.29; 1.62) | -1.54 (-1.78; -1.31) |
| **190 Hz** | -0.05 (-0.29; 0.19) | -0.79 (-1.10; -0.48) | -0.68 (-0.91; -0.45) | -0.29 (-0.66; 0.10) | -2.08 (-2.43; -1.72) | -1.53 (-1.82; -134) |
| **200 Hz** | -0.14 (-0.37; 0.11) | -0.68 (-0.99; -0.37) | -0.75 (-0.98; -0.52) | -0.30 (-0.68; 0.08) | -2.31 (-2.67; -1.94) | -1.58 (-1.82; -1.34) |
| ***Random factors σ2 (95% CrI)*** | | | | | | |
| **Vocal Category** | 0.021 (0.01; 0.04) | 0.02 (0.01; 0.05) | 0.01 (0.001; 0.02) | 0.026 (0.003; 0.10) | 0.08 (0.05; 0.12) | 0.001 (0.00006; 0.005) |

## Table S4 | Model of the auditory effect size (AES) for the different bads towards contact calls during contact call interactions.

|  | **Auditory Effect size Log (Auditory Ef. Size^2)** |
| --- | --- |
| **Fixed effects β (95% CrI)** | |
| **Intercept (M2, NB, Stack call)** | -7.74 (-8.23; -7.24) |
| **Tet call** | 0.21 (-0.17; 0.60) |
| **Treatment (B)** | 0.68 (0.11; 1.25) |
| **F3** | -0.43 (-0.86; 0.01) |
| **F1** | -2.78 (-3.09; -2.48) |
| **M3** | -0.55 (-0.86; -0.24) |
| **F2** | -0.40 (-0.78; -0.01) |
| **M1** | 0.23 (-0.11; 0.57) |
| **Tet call * Treatment (B)** | -0.87 (-1.36; -0.37) |
| **Tet call * F3** | -1.19 (-1.73; -0.65) |
| **Tet call * F1** | -0.80 (-1.35; -0.25) |
| **Tet call * M3** | -1.18 (-1.63; -0.73) |
| **Tet call * F2** | 0.03 (-0.49; 0.53) |
| **Tet call * M1** | -1.10 (-1.75; -0.45) |
| **Treatment (B) * F3** | -0.89 (-1.54; -0.25) |
| **Treatment (B) * F1** | 0.47 (0.11; 0.84) |
| **Treatment (B) * M3** | -0.41 (-0.81; 0.01) |
| **Treatment (B) * F2** | -0.14 (-0.57; 0.31) |
| **Treatment (B) * M1** | 0.18 (-0.23; 0.59) |
| **Tet call * Treatment (B) * F3** | 1.01 (0.23; 1.80) |
| **Tet call * Treatment (B) * F1** | 0.83 (0.14; 1.50) |
| **Tet call * Treatment (B) * M3** | 1.36 (0.76; 1.97) |
| **Tet call * Treatment (B) * F2** | 1.15 (0.50; 1.81) |
| **Tet call * Treatment (B) * M1** | 1.21 (0.46; 1.97) |
| **Random factors σ^2^ (95% CrI)** | |
| **Day** | 0.08 (0.05; 0.19) |
| **LFP Band** | 0.001 (0.0001; 0.003) |
| **Call category** | 0.04 (0.02; 0.06) |

## Table S5 | Effect of the breeding onset (Treatment) on the auditory effect size (AES) of males and females towards tet and stack contact calls of their mates.


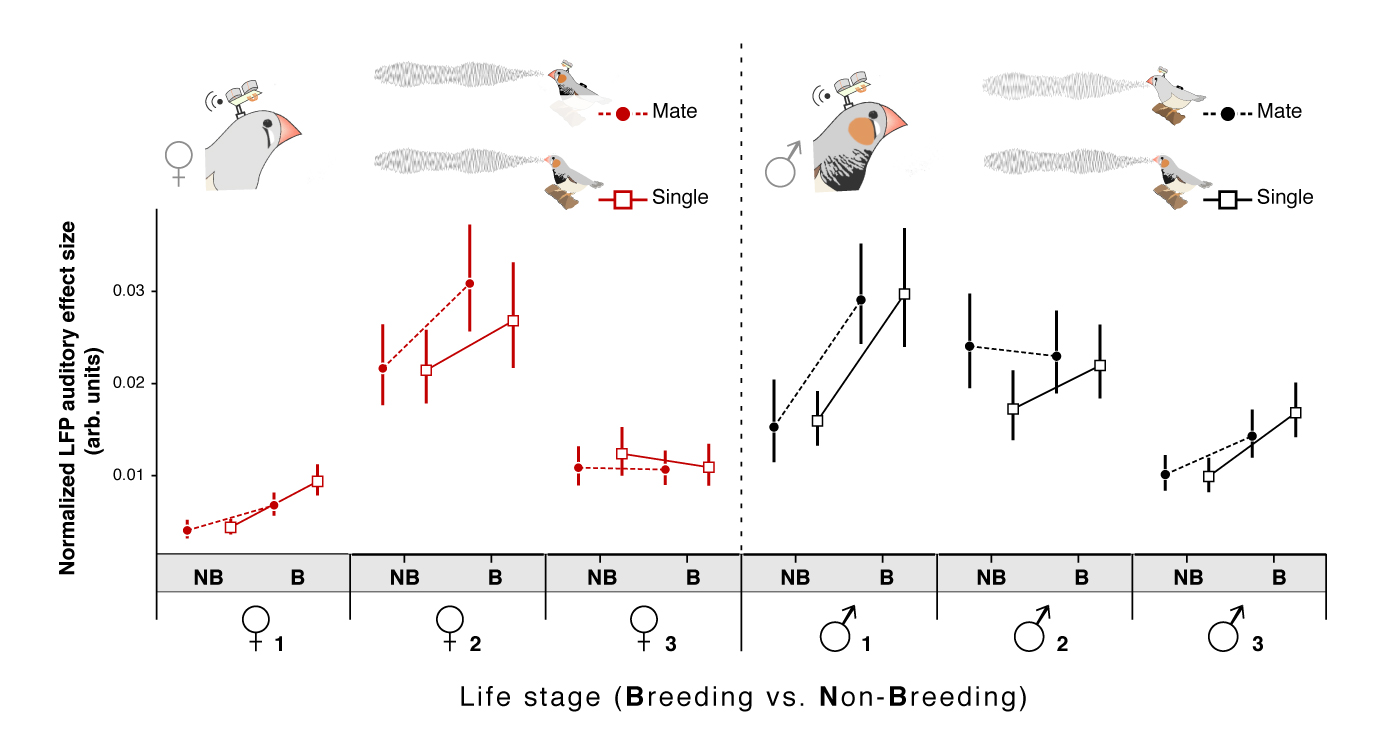


## Figure S6 | Females’ (left) and males’ (right) auditory local field potential response amplitude (for the 30-50 Hz band) in response to the second most frequent contact call (tet call) of their mate (circles and dotted line) and the single male (squares and solid lines) in non-breeding (NB) and breeding (B) conditions. Shapes represent the estimated mean from the model and vertical bars the 95 % CrI.

##
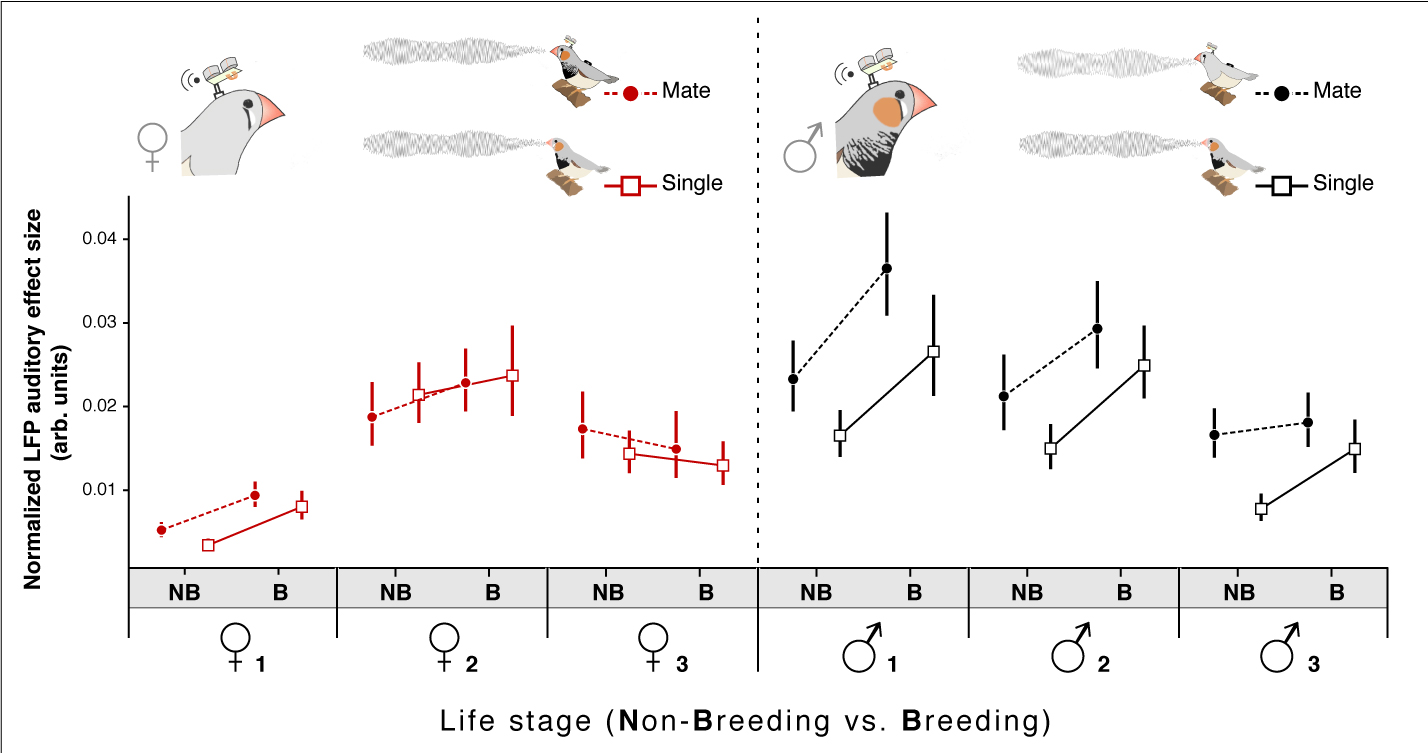


## Figure S7 | Females’ (left) and males’ (right) auditory local field potential response amplitude (for the 30-50 Hz band) in response to the most frequent contact call (stack call) of their mate (circles and dotted line) and the single male (squares and solid lines) in non-breeding (NB) and breeding (B) conditions. Shapes represent the estimated mean from the model and vertical bars the 95 % CrI.

|  | **Auditory Effect size Log (Auditory Ef. Size^2) to Stack Calls** | **Auditory Effect size Log (Auditory Ef. Size^2) to Tet Calls** |
| --- | --- | --- |
| **Fixed effects β (95% CrI)** | | |
| **Intercept (M2, NB, Mate call)** | -7.703 (-8.123; -7.285) | -7.450 (-7.874; -7.034) |
| **Single Male** | -0.696 (-1.016; -0.374) | -0.669 (-1.043; -0.299) |
| **Treatment (B)** | 0.644 (0.217; 1.069) | -0.099 (-0.618; 0.428) |
| **F3** | -0.406 (-0.834; 0.021) | -1.589 (-1.906; -1.269) |
| **F1** | -2.799 (-3.097; -2.050) | -3.556 (-3.988; -3.119) |
| **M3** | -0.490 (-0.792; -0.181) | -1.733 (-2.038; -1.421) |
| **F2** | -0.251 (-0.613; 0.115) | -0.211 (-0.533; 0.114) |
| **M1** | 0.183 (-0.140; 0.508) | -0.909 (-1.427; -0.401) |
| **Single Malel * Treatment (B)** | 0.372 (-0.025; 0.766) | 0.582 (0.091; 1.079) |
| **Single Male * F3** | 0.321 (-0.158; 0.798) | 0.930 0.451; 1.399) |
| **Single Male * F1** | -0.148 (-0.563; 0.278) | 0.828 (0.290; 1.374) |
| **Single Male * M3** | -0.821 (-1.262; -0.397) | 0.628 (0.184; 1.069) |
| **Single Male * F2** | 0.961 (0.562; 1.371) | 0.652 (0.204; 1.091) |
| **Single Male * M1** | 0.010 (-0.352; 0.383) | 0.757 (0.158; 1.364) |
| **Treatment (B) * F3** | -0.944 (-1.582; -0.312) | 0.058 (-0.381; 0.492) |
| **Treatment (B) * F1** | 0.522 (0.161; 0.868) | 1.123 (0.578; 1.658) |
| **Treatment (B) * M3** | -0.473 (-0.880; -0.079) | 0.788 (0.350; 1.226) |
| **Treatment (B) * F2** | -0.250 (-0.671; 0.166) | 0.804 (0.348; 1.265) |
| **Treatment (B) * M1** | 0.256 (-0.141; 0.643) | 1.381 (0.776;1.985) |
| **Single Male * Treatment (B) * F3** | -0.283 (-0.999; 0.431) | -0.797 (-1.454; -0.139) |
| **Single Male * Treatment (B) * F1** | 0.159 (-0.406; 0.727) | -0.095 (-0.769; 0.569) |
| **Single Male * Treatment (B) * M3** | 0.760 (0.162; 1.368) | -0.214 (-0.815; 0.380) |
| **Single Male* Treatment (B) * F2** | -0.559 (-1.120; 0.019) | -0.848 (-1.514; -0.179) |
| **Single Male * Treatment (B) * M1** | -0.324 (-0.879; 0.243) | -0.618 (-1.375; 0.139) |
| **Random factors σ^2^ (95% CrI)** | | |
| **Day** | 0.031 (0.017; 0.067) | 0.062 (0.038; 0.114) |
| **LFP Band** | 0.001 (0.000; 0.004) | 0.001 (0.000; 0.004) |
| **Call category** | 0.044 (0.026; 0.067) | 0.023 (0.010; 0.042) |

## Table S6 | Effect of the breeding onset (Treatment) on the auditory effect size (AES) of males and females towards: i) Stack contact calls of the mate and the single male; ii) Tet contact calls of the mate and the single male during daytime interactions.


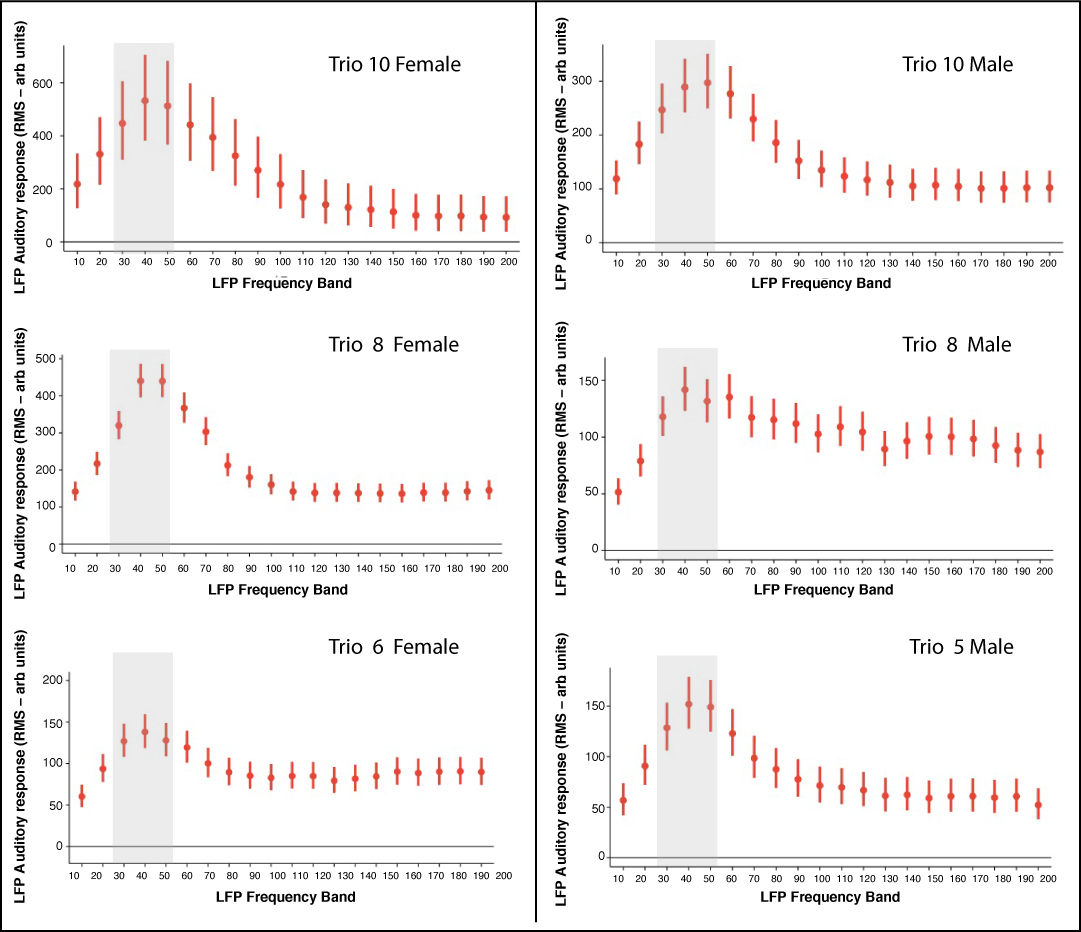


## Figure S8 | Average auditory amplitude modulation (Auditory-Baseline) from each individual during the night playbacks. As in the day recordings the band of 30-50 Hz (marked grey) is the one with the highest amplitude modulation.

|  | **Auditory Effect size (AES)** |
| --- | --- |
| **Fixed effects β (95% CrI)** | |
| **Intercept (F, NB, Distance Calll)** | 0.276 (0.140; 0.412) |
| **Kackle call** | -0.102 (-0.193; -0.014) |
| **Stack call** | 0.045 (-0.043; 0.0131) |
| **Tet Call** | 0.114 (0.028; 0.202) |
| **Whine Call** | -0.059(-0.157; 0.004) |
| **White Noise** | 0.090 (0.002; 0.177) |
| **Treatment (B)** | 0.137 (0.023; 0.253) |
| **Sex (M)** | -0.026 (-0.185; 0.134) |
| **Kackle*Treatment** | 0.030 (-0.094; 0.155) |
| **Stack*Treatment** | -0.002 (-0.122; 0.123) |
| **Tet*Treatment** | 0.130 (0.006; 0.255) |
| **Whine*Treatment** | -0.091 (-0.229; 0.049) |
| **WhiteNoise*Treatment** | -0.072 (-0.195; 0.054) |
| **Kackle*Sex** | 0.344 (0.213; 0.476) |
| **Stack*Sex** | -0.034 (-0.159; 0.089) |
| **Tet*Sex** | -0.049 (-0.175; 0.076) |
| **Whine*Sex** | -0.089 (-0.229; 0.050) |
| **WhiteNoise*Sex** | 0.114 (-0.009; 0.239) |
| **Treatment*Sex** | -0.173 (-0.298; -0.044) |
| **Kackle*Sex*Treatment** | 0.015 (-0.174; 0.020) |
| **Stack*Sex*Treatment** | -0.004 (-0.178; 0.173) |
| **Tet*Sex*Treatment** | -0.116 (-0.293; 0.057) |
| **Whine*Sex*Treatment** | 0.113 (-0.085; 0.308) |
| **WhiteNoise*Sex*Treatment** | 0.153 (-0.024; 0.329) |
| **Random factors σ^2^ (95% CrI)** | |
| **Day** | 0.002 (0.001; 0.005) |
| **LFP Band** | 0.002 (0.000; 0.005) |
| **ID** | 0.007 (0.003; 0.014) |

## Table S7 | Effect of the breeding onset (Treatment) on the auditory effect size (AES) of males and females towards the different contact calls of their mate during nighttime playbacks.

|  | **Auditory Effect size (AES) towards the song** |
| --- | --- |
| **Fixed effects β (95% CrI)** | |
| **Intercept (F, NB, Song)** | 0.157 (0.0601; 0.254) |
| **Treatment (B)** | -0.044 (-0.164; 0.077) |
| **Random factors σ^2^ (95% CrI)** | |
| **Day** | 0.0053 (0.0025; 0.0135) |
| **LFP Band** | 0.00 (0.00; 0.00) |
| **ID** | 0.001 (0.0006; 0.0038) |

## Table S8 | Effect of the breeding onset (Treatment) on the auditory effect size (AES) of females towards the song of their mate during nighttime playbacks.

|  | **Kackle Call** | **Tet Call** | **Distance Call** | **Stack Call** |
| --- | --- | --- | --- | --- |
|  | **Auditory Effect size (AES)** | **Auditory Effect size (AES)** | **Auditory Effect size (AES)** | **Auditory Effect size (AES)** |
| **Fixed effects β (95% CrI)** | | | | |
| **Intercept (NB, Unfamiliar Male)** | 0.184 (0.078; 0.292) | 0.287 (0.115; 0.455) | 0.166 (0.081; 0.251) | 0.210 (0.105;0.313) |
| **Treatment (B)** | 0.137 (0.055; 0.219) | 0.098 (-0.002; 0.199) | -0.036 (-0.121; 0.051) | 0.084 (-0.017; 0.186) |
| **Single Male** | 0.070 (-0.025; 0.164) | 0.130 (0.030; 0.230) | 0.093 (0.006; 0.181) | 0.029 (-0.074; 0.133) |
| **Mate Male** | 0.005 (-0.081; 0.088) | 0.117 (0.017; 0.220) | 0.126 (0.040; 0.212) | 0.127 (0.024; 0.229) |
| **Own call** | 0.213 (0.119; 0.308) | 0.009 (-0.090; 0.106) | 0.03 (-0.055; 117) | -0.034 (-0.137; 0.066) |
| **Unfamiliar Female** | -0.014 (-0.098; 0.069) | -0.007 (-0.106; 0.094) | -0.083 (-0.171; 0.002) | -0.234 (-0.337; -0.132) |
| **Treatment*Single Male** | 0.019 (-0.114; 0.150) | 0.101 (-0.041; 0.242) | 0.106 (-0.017; 0.229) | 0.009 (-0.137; 0.152) |
| **Treatment*Mate Male** | -0.012 (-0.130; 0.108) | 0.127 (-0.018; 0.265) | 0.132 (0.009; 0.254) | 0.007 (-0.137; 0.151) |
| **Treatment*Own Call** | 0.027 (-0.108; 0.159) | -0.025 (-0.169; 0.114) | -0.011 (-0.134; 0.107) | -0.083 (-0.229; 0.059) |
| **Treatment*Unfamiliar Female** | 0.012 (-0.105; 0.130) | 0.012 (-0.131; 0.158) | -0.013 (-0.137; 0.111) | 0.011 (-0.133; 0.156) |
| **Random factors σ^2^ (95% CrI)** | | | | |
| **LFP Band** | 0.001 (0.000; 0.002) | 0.001 (0.000; 0.004) | 0.001 (0.000; 0.004) | 0.0003 (0.00001; 0.002) |
| **ID** | 0.005 (0.001; 0.021) | 0.019 (0.010; 0.053) | 0.002 (0.000; 0.006) | 0.004 (0.001; 0.013) |

## Table S9 | Effect of the breeding onset (Treatment) on the auditory effect size (AES) of females towards the kackle, stack, tet and distance calls from different during nighttime playbacks.


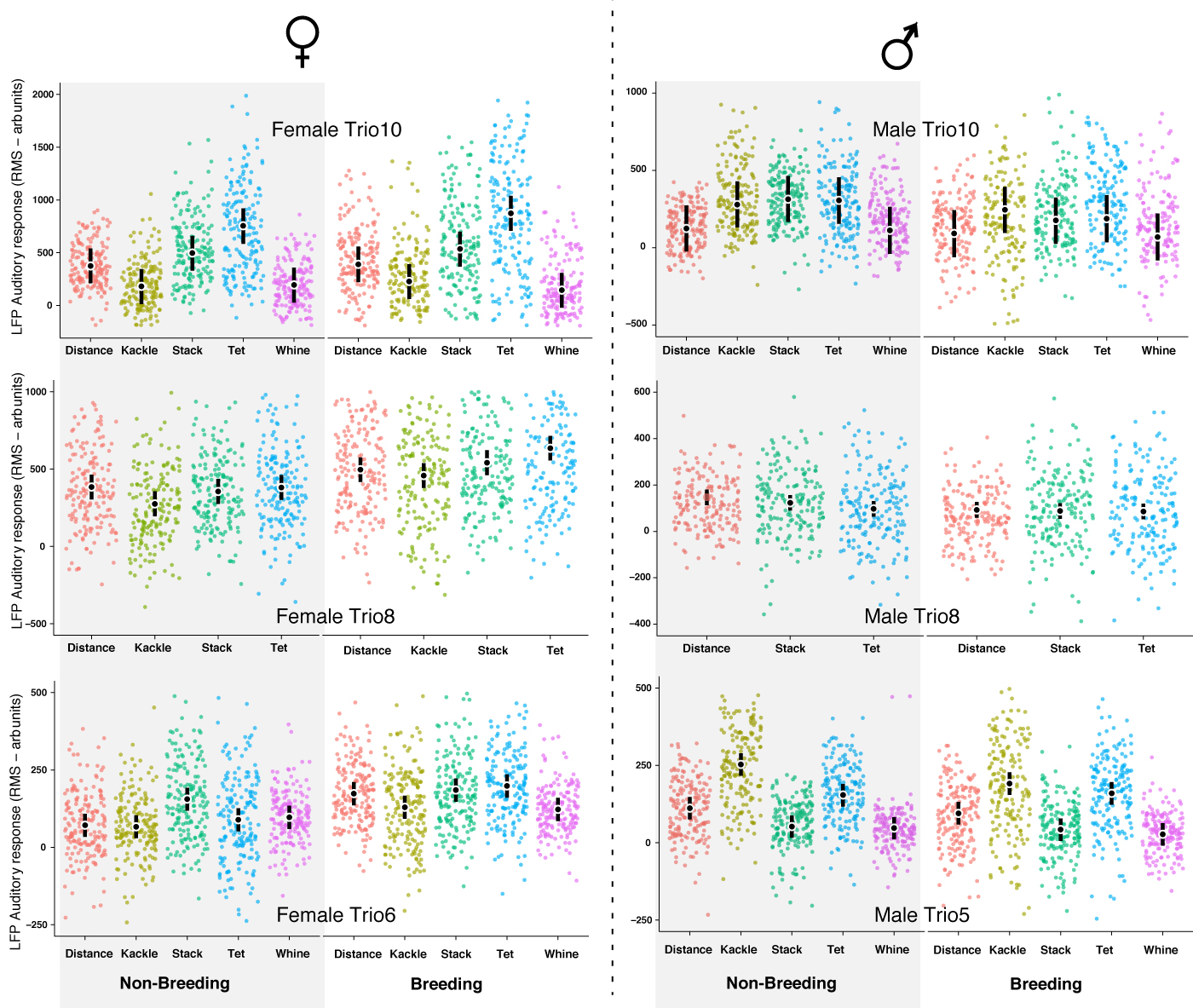


**Figure S9 |** Individual females’ (left) and males’ (right) auditory local field potential response amplitude (for the 30-50 Hz band) in response to the different calls of their mate (circles and dotted line) in non-breeding (NB) and breeding (B) conditions during night playbacks. Shapes represent the estimated mean from the model, vertical bars the 95 % CrI and coloured dots the raw data.

|  | **Female Trio10** | **Female Trio8** | **Female Trio6** | **Male Trio10** | **Male Trio8** | **Male Trio5** |
| --- | --- | --- | --- | --- | --- | --- |
|  | **Auditory Effect size (AES)** | **Auditory Effect size (AES)** | **Auditory Effect size (AES)** | **Auditory Effect size (AES)** | **Auditory Effect size (AES)** | **Auditory Effect size (AES)** |
| **Fixed effects β (95% CrI)** | | | | | | |
| **Intercept (NB, DistanceCall)** | 368.670 (191.909; 545.925) | 406.314 (298.150; 511.942) | 71.650 (33.994; 108.728) | 142.623 (-25.448; 309.563) | 135.868 (109.604; 162.483) | 106.990 (68.951; 145.055) |
| **Treatment (B)** | 14.571 (-236.718; 253.586) | 86.375 (22.389; 150.326) | 101.246 (49.043; 153;416) | -58.568 (-295.415; 174.683) | -59.514 (-96.506; -21.448) | 2.300 (-48.461; 53.874) |
| **Kackle call** | -180.899 (-258.531;-102.394) | -142.811 (-204.387; -80.858) | -5.125 (-28.190; 17.999) | 101.704 (-24.568; 225.104) | - | 146.378 (121.898; 171.219) |
| **Stack call** | 142.967 (65.775; 221.441) | -115.531 (-177.916; -55.451) | 84.808 (61.651; 108.147) | 154.904 (28.682; 282.755) | -14.569 (-49.256; 20.186) | -61.906 (-86.517; -37.529) |
| **Tet Call** | 392.256 (315.209; 469.778) | - | 17.849 (-5.293; 41.829) | 131.000 (7.014; 259.255) | -47.518 (-82.047 (-12.810) | 38.618 (13.733; 63.409) |
| **Wine Call** | -157.026 (-235.003; -79.160) | -42.309 (-104.087; 19.227) | 25.865 (2.396; 49.451) | -43.134 (-168.788; 83.375) | - | -67.011 (-91.859; -42.863) |
| **Treatment*Kackle** | 22.322 (-90.095; 131.187) | 81.354 (-6.169; 169.893) | -39.207 (-71.677; -6.252) | 91.018 (-88.117; 269.868) | - | -36.777 (-71.682; -2.253) |
| **Treatment*Stack** | -6.745 (-115.556; 102.863) | 94.896 (10.963; 182.490) | -73.274 (-107.283;-40.041) | -69.337 (-246.321; 107.749) | 46.632 (-2.412; 94.974) | -8.250 (-42.621; 26.141) |
| **Treatment*Tet** | 61.310 (-49.394; 170.055) | 236.308 (149.213; 322.906) | 7.189 (-25.520; 39.952) | -73.073 (-251.456; 107.182) | 67.713 (18.782; 116.550) | 0.964 (-33.497; 36.137) |
| **Treatment*Whine** | -53.043 (-164.697; 57.3158) | - | -76.927 (-110.167; -43.396) | 13.35 (-164.63; 192.876) | - | -8.525 (-43.491; 26.010) |
| **Random factors σ^2^ (95% CrI)** | | | | | | |
| **LFP Band** | 563.1 (56.7; 1707.2) | 7089.1 (4330.6; 10609.1) | 11.074 (0.439; 50.553) | 783.26 (41.536; 2854.492) | 0 (0;0) | 121.27 (25.55; 294.069) |
| **Day** | 9792 (2944.1; 46368) | 69.407 (6.138; 269.171) | 432.82 (146.94; 1715.5) | 8866 (3270; 23876) | 42.15 (3.64; 181.053) | 404.662 (138.75; 1556.80) |

## Table S10 | Effect of the breeding onset (Treatment) on the auditory effect size (AES) of every individual towards the kackle, stack, tet whine and distance calls of their mate during nighttime playbacks.

**
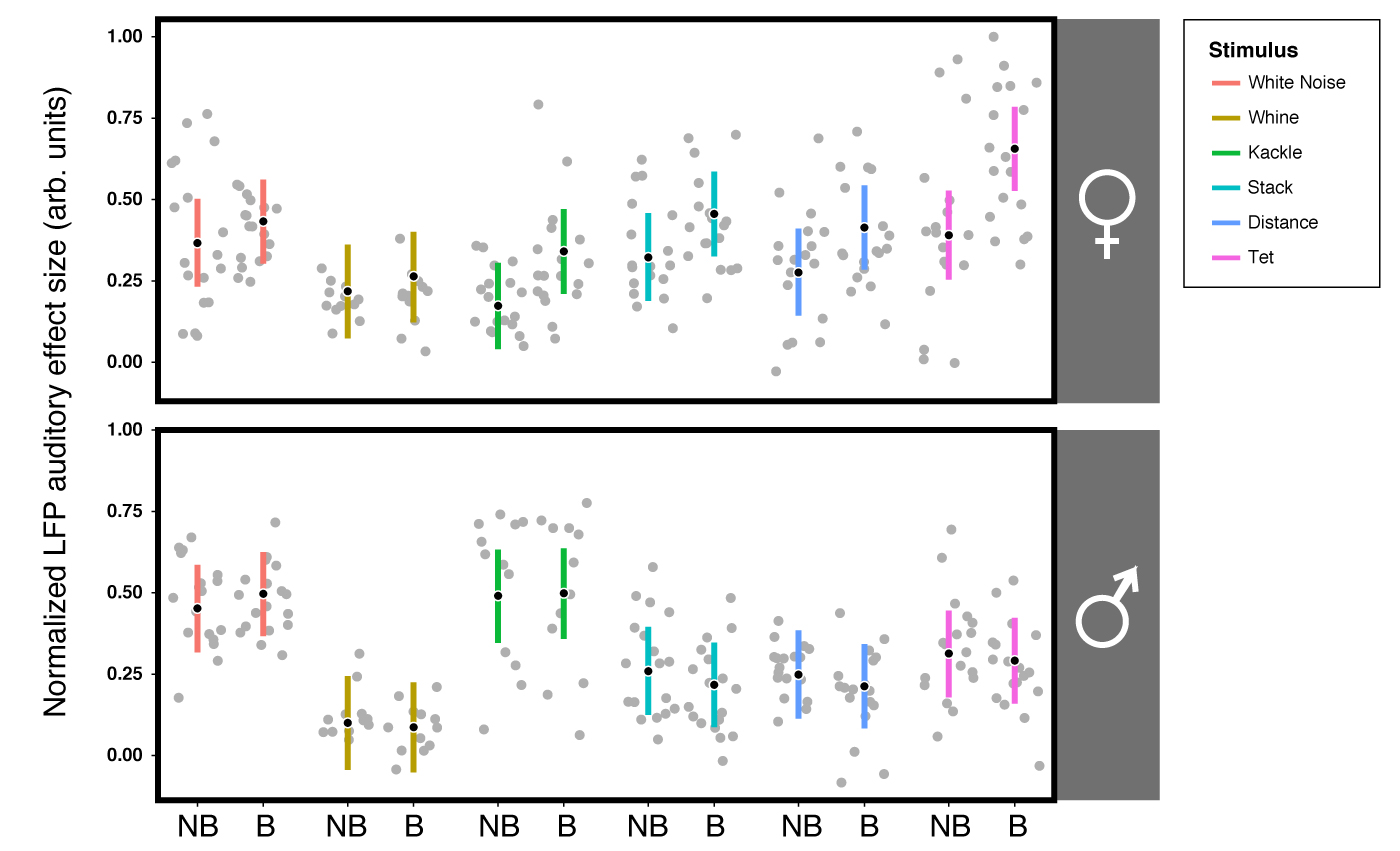
**

**Figure S10 |** Version of figure 4 C with the addition of the raw data points (grey dots).
